# Supplementary material for: Hybrid Models and Biological Model Reduction with PyDSTool
Source: PLoS Comput Biol. 2012 Aug 9;8(8):e1002628. doi: 10.1371/journal.pcbi.1002628 (PMC3415397; doi:10.1371/journal.pcbi.1002628)
Supplement: Text S4 — Complete source code for the PyDSTool package (version 0.88.120504). Includes API documentation and help files linking to web pages. This file is identical to the current public release on Sourceforge.net. (ZIP) [file pcbi.1002628.s004.zip › PyDSTool/html/PyDSTool.Points-pysrc.html]

xml version="1.0" encoding="ascii"?


PyDSTool.Points


| Home | Trees | Indices | Help | PyDSTool |
| --- | --- | --- | --- | --- |

Package PyDSTool ::
Module Points

[hide private]

[frames] | no frames]

# Source Code for Module PyDSTool.Points

```
   1  """Point and Pointset enhanced array classes.
 
   2  
 
   3  (Objects of both classes are mutable.)
 
   4  
 
   5      Robert Clewley, February 2006
 
   6  """ 
   7  
 
   8  # ----------------------------------------------------------------------------
 
   9  
 
  10  from __future__ import division 
  11  
 
  12  ## PyDSTool imports
 
  13  from utils import * 
  14  from common import * 
  15  from errors import * 
  16  from parseUtils import symbolMapClass, mapNames 
  17  
 
  18  ## Other imports
 
  19  from numpy import Inf, NaN, isfinite, array2string, r_, c_, \
 
  20      less, greater, linalg, shape, array, argsort, savetxt, \
 
  21      take, zeros, transpose, resize, indices, concatenate, rank 
  22  
 
  23  from numpy import complex, complexfloating, int, integer, \
 
  24       float, floating, float64, complex128, int32 
  25  from numpy import any, all, alltrue, sometrue, ndarray 
  26  
 
  27  
 
  28  import sys 
  29  from copy import copy, deepcopy 
  30  
 
  31  
 
  32  __all__ = ['Point', 'Pointset', 'isparameterized', 'pointsToPointset',
 
  33             'PointInfo', 'makeNonParameterized', 'arrayToPointset',
 
  34             'VarCaller', 'comparePointCoords', 'importPointset',
 
  35             'exportPointset', 'mergePointsets', 'padPointset',
 
  36             'export_pointset_to_CSV'] 
  37  
 
  38  #----------------------------------------------------------------------------
 
  39  
 
  40  
 


41 -class VarCaller(object):


42      """Wrapper for Variable type to call Pointset and return array type.""" 
  43  
 


44 -    def __init__(self, pts):


45          if isinstance(pts, (Point, Pointset)): 
  46              self.pts = pts 
  47          else: 
  48              raise TypeError("Invalid type for pts argument")

  49  
 


50 -    def __call__(self, x):


51          return self.pts(x).toarray()

  52  
 
  53  
 
  54  #----------------------------------------------------------------------------
 
  55  
 
  56  
 
  57  # for internal use
 
  58  point_keys = ['coorddict', 'coordarray', 'coordtype', 'norm', 'labels'] 
  59  
 
  60  
 


61 -class Point(object):


62      """N-dimensional point class.""" 
  63  
 
  64      # Contains an ordered list of names for the coordinates (to
 
  65      # suggest how the points belong to a particular space specified
 
  66      # using a particular basis)
 


67 -    def __init__(self, kwd=None, **kw):


68          if kwd is not None: 
  69              if kw != {}: 
  70                  raise ValueError("Cannot mix keyword dictionary and keywords") 
  71              kw = kwd 
  72          self._parameterized = False 
  73          self.labels = {} 
  74          if intersect(kw.keys(), point_keys) == []: 
  75              # creating Point from dictionary
 
  76              temp_kw = {} 
  77              temp_kw['coorddict'] = copy(kw) 
  78              kw = copy(temp_kw) 
  79          if 'coorddict' in kw: 
  80              coorddict = {} 
  81              try: 
  82                  ct = kw['coordtype'] 
  83              except KeyError: 
  84                  self.coordtype = float 
  85              else: 
  86                  try: 
  87                      self.coordtype = _num_equivtype[ct] 
  88                  except KeyError: 
  89                      raise TypeError('Coordinate type %s not valid for Point'%str(ct)) 
  90              for c, v in kw['coorddict'].iteritems(): 
  91                  if not isinstance(c, str): 
  92                      c_key = repr(c) 
  93                  else: 
  94                      c_key = c 
  95                  if isinstance(v, list): 
  96                      coorddict[c_key] = array(v, self.coordtype) 
  97                  elif isinstance(v, ndarray): 
  98                      if len(v) == 1: 
  99                          coorddict[c_key] = v[0] 
 100                      else: 
 101                          coorddict[c_key] = array(v) 
 102                      assert compareNumTypes(self.coordtype, coorddict[c_key].dtype.type), \
 
 103                             'type mismatch' 
 104                  elif isinstance(v, _float_types): 
 105                      assert compareNumTypes(self.coordtype, _float_types), \
 
 106                             'type mismatch' 
 107                      coorddict[c_key] = array([v], self.coordtype) 
 108                  elif isinstance(v, _int_types): 
 109                      assert compareNumTypes(self.coordtype, _real_types), \
 
 110                             'type mismatch' 
 111                      coorddict[c_key] = array([v], self.coordtype) 
 112  ##                elif isinstance(v, _complex_types):
 
 113  ##                    assert compareNumTypes(self.coordtype, complex), 'type mismatch'
 
 114  ##                    coorddict[c_key] = array([v], self.coordtype)
 
 115                  else: 
 116                      raise TypeError("Must pass numeric type or sequence of "
 
 117                                      "numeric types") 
 118              self.coordnames = coorddict.keys() 
 119              # only way to order dictionary keys for array is to sort
 
 120              self.coordnames.sort() 
 121              self.dimension = len(self.coordnames) 
 122              datalist = [] 
 123              for c in self.coordnames: 
 124                  assert not isinstance(coorddict[c], (list, tuple)), 'type mismatch' 
 125                  datalist.append(coorddict[c][0]) 
 126              self.coordarray = array(datalist, self.coordtype) 
 127              r = rank(self.coordarray) 
 128              if r == 1: 
 129                  pass 
 130              elif r == 0: 
 131                  self.coordarray = self.coordarray.ravel() 
 132              else: 
 133                  raise ValueError("Invalid rank for coordinate array: %i"%r) 
 134              assert self.dimension == self.coordarray.shape[0], "Invalid coord array" 
 135          elif 'coordarray' in kw: 
 136              # 'coordtype' key is optional unless 'array' is actually a list,
 
 137              # when this key specifies the internal Python to use
 
 138              if isinstance(kw['coordarray'], ndarray): 
 139                  # use 'array' constructor to ensure that copy is made of array
 
 140                  # in case either original or new array is independently changed.
 
 141                  array_temp = array(kw['coordarray']) 
 142                  try: 
 143                      self.coordtype = _num_equivtype[array_temp.dtype.type] 
 144                  except KeyError: 
 145                      raise TypeError('Coordinate type %s not valid for Point'%str(ct)) 
 146              elif isinstance(kw['coordarray'], list): 
 147                  try: 
 148                      self.coordtype = _num_equivtype[kw['coordtype']] 
 149                  except KeyError: 
 150                      raise TypeError('Coordinate type %s not valid for Point'%str(ct)) 
 151                  array_temp = array(kw['coordarray'], self.coordtype) 
 152              else: 
 153                  raise TypeError('Coordinate type %s not valid for Point'%str(type(kw['coordarray']))) 
 154              r = rank(array_temp) 
 155              if r == 1: 
 156                  self.coordarray = array_temp 
 157              elif r == 0: 
 158                  self.coordarray = array_temp.ravel() 
 159              else: 
 160                  raise ValueError("Invalid rank for coordinate array: %i"%r) 
 161              self.dimension = self.coordarray.shape[0] 
 162              if 'coordnames' in kw: 
 163                  if isinstance(kw['coordnames'], str): 
 164                      coordnames = [kw['coordnames']] 
 165                  else: 
 166                      coordnames = kw['coordnames'] 
 167              else: 
 168                  coordnames = [str(cix) for cix in range(self.dimension)] 
 169              if len(coordnames) != self.dimension: 
 170                  print "Point initialization error:" 
 171                  print "Found coord names: ", coordnames, \
 
 172                               "(dimension = %s)"%len(coordnames) 
 173                  print "vs. data dimension =", self.dimension 
 174                  raise ValueError("Mismatch between number of coordnames and "
 
 175                                   "dimension of data") 
 176              cs = array(coordnames) 
 177              order = cs.argsort() 
 178              self.coordnames = cs[order].tolist() 
 179              self.coordarray = self.coordarray[order] 
 180          else: 
 181              raise ValueError("Missing coord info in keywords") 
 182          assert isUniqueSeq(self.coordnames), 'Coordinate names must be unique' 
 183          self.makeIxMaps() 
 184          if 'norm' in kw: 
 185              if kw['norm'] == 0: 
 186                  raise ValueError("Norm order for point cannot be zero") 
 187              self._normord = kw['norm'] 
 188          else: 
 189              self._normord = 2 
 190          # extra information (for special bifurcation point data)
 
 191          if 'labels' in kw: 
 192              self.addlabel(kw['labels'])

 193  
 
 194  
 


195 -    def mapNames(self, themap):


196          """Map coordinate names and label(s), using a symbol
 
 197          map of class symbolMapClass.""" 
 198          new_coordnames = array(themap(self.coordnames)) 
 199          assert isUniqueSeq(new_coordnames.tolist()), 'Coordinate names must be unique' 
 200          order = argsort(new_coordnames) 
 201          self.coordarray = self.coordarray[order] 
 202          self.coordnames = new_coordnames[order].tolist() 
 203          self.makeIxMaps() 
 204          # the following call will be inherited by Pointset, and
 
 205          # works on Point labels-as-dict and Pointset labels-as-
 
 206          # PointInfo objects, as the latter have their own
 
 207          # mapNames method which will get called.
 
 208          self.labels = mapNames(themap, self.labels)

 209  
 
 210  
 


211 -    def addlabel(self, label):


212          if label is None: 
 213              pass 
 214          elif isinstance(label, str): 
 215              self.labels = {label: {}} 
 216          elif isinstance(label, tuple) and len(label)==2: 
 217              if isinstance(label[0], str) and isinstance(label[1], dict): 
 218                  self.labels[label[0]] = label[1] 
 219          elif isinstance(label, dict): 
 220              self.labels = label 
 221          else: 
 222              raise TypeError("Point label must be a string, a pair, or a dict")

 223  
 
 224  
 


225 -    def removelabel(self):


226          self.labels = {}

 227  
 
 228  
 


229 -    def makeIxMaps(self):


230          self._name_ix_map = dict(zip(self.coordnames, range(self.dimension))) 
 231          self._ix_name_map = copy(self.coordnames)

 232  
 
 233  
 


234 -    def todict(self, aslist=False):


235          """Convert Point to a dictionary of array values (or of list with aslist=True).""" 
 236          if aslist: 
 237              return dict(zip(self._ix_name_map, self.coordarray.tolist())) 
 238          else: 
 239              return dict(zip(self._ix_name_map, self.coordarray))

 240  
 


241 -    def __contains__(self, coord):


242          return coord in self.coordnames

 243  
 


244 -    def __delitem__(self, k):


245          raise NotImplementedError

 246  
 


247 -    def get(self, coord, d=None):


248          if coord in self.coordnames: 
 249              return self.__call__(coord) 
 250          else: 
 251              return d

 252  
 


253 -    def update(self, d):


254          for k, v in d.iteritems(): 
 255              self.coordarray[self._map_names_to_ixs(k)] = v

 256  
 


257 -    def items(self):


258          return zip(self._ix_name_map, self.coordarray)

 259  
 


260 -    def iteritems(self):


261          return iter(zip(self._ix_name_map, self.coordarray))

 262  
 


263 -    def values(self):


264          return self.coordarray.tolist()

 265  
 


266 -    def itervalues(self):


267          return iter(self.coordarray.tolist())

 268  
 


269 -    def keys(self):


270          return self._ix_name_map

 271  
 


272 -    def iterkeys(self):


273          return iter(self._ix_name_map)

 274  
 


275 -    def has_key(self, k):


276          return k in self.coordnames

 277  
 
 278  
 


279 -    def _map_names_to_ixs(self, namelist):


280          try: 
 281              try: 
 282                  # single string
 
 283                  return self._name_ix_map[namelist] 
 284              except TypeError: 
 285                  # list of strings
 
 286                  return [self._name_ix_map[n] for n in namelist] 
 287          except KeyError, e: 
 288              raise PyDSTool_KeyError("Name not found: "+str(e))

 289  
 
 290  
 


291 -    def __len__(self):


292          return self.dimension

 293  
 
 294  
 


295 -    def _force_coords_to_ixlist(self, x):


296          if x is None: 
 297              return range(self.dimension) 
 298          elif x in xrange(self.dimension): 
 299              # only used for recursive calls
 
 300              return [x] 
 301          elif x in self.coordnames: 
 302              # only used for recursive calls
 
 303              return [self._name_ix_map[x]] 
 304          elif isinstance(x, _seq_types): 
 305              if len(x) == 0: 
 306                  return range(self.dimension) 
 307              else: 
 308                  return [self._force_coords_to_ixlist(el)[0] for el in x] 
 309          elif isinstance(x, slice): 
 310              stop = x.stop or self.dimension 
 311              s1, s2, s3 = x.indices(stop) 
 312              if s1 < 0 or s2 > self.dimension or s1 >= self.dimension: 
 313                  raise ValueError("Slice index out of range") 
 314              return range(s1, s2, s3) 
 315          else: 
 316              raise ValueError("Invalid coordinate / index: %s"%str(x) + \
 
 317                               " -- coord names are: %s"%str(self.coordnames))

 318  
 
 319  
 


320 -    def __call__(self, coords):


321          if coords in xrange(self.dimension+1): 
 322              if coords == self.dimension: 
 323                  # trap for when Point is used as an iterator, i.e. as
 
 324                  # for x in pt -- avoids writing an __iter__ method that
 
 325                  # will be inherited by Pointset, which already iterates fine
 
 326                  raise StopIteration 
 327              else: 
 328                  return self.coordarray[coords] 
 329          elif coords in self.coordnames: 
 330              ix = self._name_ix_map[coords] 
 331              return self.coordarray[ix] 
 332          else: 
 333              ixlist = self._force_coords_to_ixlist(coords) 
 334              return Point({'coordarray': self.coordarray[ixlist],
 
 335                        'coordnames': [self.coordnames[i] for i in ixlist],
 
 336                        'coordtype': self.coordtype,
 
 337                        'norm': self._normord,
 
 338                        'labels': self.labels})

 339  
 
 340      __getitem__ = __call__ 
 341  
 
 342  #    def __iter__(self):
 
 343  #        return self.coordarray.__iter__()
 
 344  
 
 345  
 


346 -    def __setitem__(self, ixarg, val):


347          """Change coordinate array values.""" 
 348          ixs = self._force_coords_to_ixlist(ixarg) 
 349          if len(ixs) == 1: 
 350              val = [val] 
 351          try: 
 352              for i, v in zip(ixs,val): 
 353                  self.coordarray[i] = v 
 354          except TypeError: 
 355              raise TypeError("Bad value type for Point")

 356  
 
 357  
 


358 -    def toarray(self):


359          if self.dimension == 1: 
 360              return self.coordarray[0] 
 361          else: 
 362              return self.coordarray

 363  
 
 364  
 


365 -    def __add__(self, other):


366          res = self.copy() 
 367          try: 
 368              res.coordarray += other.coordarray 
 369          except AttributeError: 
 370              res.coordarray += other 
 371          return res

 372  
 
 373      __radd__ = __add__ 
 374  
 


375 -    def __sub__(self, other):


376          res = self.copy() 
 377          try: 
 378              res.coordarray -= other.coordarray 
 379          except AttributeError: 
 380              res.coordarray -= other 
 381          return res

 382  
 


383 -    def __rsub__(self, other):


384          res = self.copy() 
 385          try: 
 386              res.coordarray = other.coordarray - res.coordarray 
 387          except AttributeError: 
 388              res.coordarray = other - res.coordarray 
 389          return res

 390  
 


391 -    def __mul__(self, other):


392          res = self.copy() 
 393          try: 
 394              res.coordarray *= other.coordarray 
 395          except AttributeError: 
 396              res.coordarray *= other 
 397          return res

 398  
 
 399      __rmul__ = __mul__ 
 400  
 


401 -    def __div__(self, other):


402          res = self.copy() 
 403          try: 
 404              res.coordarray /= other.coordarray 
 405          except AttributeError: 
 406              res.coordarray /= other 
 407          return res

 408  
 
 409      __truediv__ = __div__ 
 410  
 


411 -    def __rdiv__(self, other):


412          res = self.copy() 
 413          try: 
 414              res.coordarray = other.coordarray / res.coordarray 
 415          except AttributeError: 
 416              res.coordarray = other / res.coordarray 
 417          return res

 418  
 
 419      __rtruediv__ = __rdiv__ 
 420  
 


421 -    def __pow__(self, other):


422          res = self.copy() 
 423          res.coordarray **= other 
 424          return res

 425  
 


426 -    def __neg__(self):


427          res = self.copy() 
 428          res.coordarray = - res.coordarray 
 429          return res

 430  
 


431 -    def __pos__(self):


432          return self.copy()

 433  
 


434 -    def __lt__(self, other):


435          try: 
 436              assert shape(self) == shape(other) 
 437              if hasattr(other, 'coordnames'): 
 438                  if self.coordnames != other.coordnames: 
 439                      raise ValueError("Coordinate mismatch") 
 440              return linalg.norm(self.coordarray, self._normord) < \
 
 441                     linalg.norm(other.coordarray, self._normord) 
 442          except (AttributeError, TypeError, AssertionError): 
 443              return self.coordarray < other 
 444          except ZeroDivisionError: 
 445              raise ValueError("Norm order for point cannot be zero")

 446  
 


447 -    def __gt__(self, other):


448          try: 
 449              assert shape(self) == shape(other) 
 450              if hasattr(other, 'coordnames'): 
 451                  if self.coordnames != other.coordnames: 
 452                      raise ValueError("Coordinate mismatch") 
 453              return linalg.norm(self.coordarray, self._normord) > \
 
 454                     linalg.norm(other.coordarray, self._normord) 
 455          except (AttributeError, TypeError, AssertionError): 
 456              return self.coordarray > other 
 457          except ZeroDivisionError: 
 458              raise ValueError("Norm order for point cannot be zero")

 459  
 


460 -    def __le__(self, other):


461          try: 
 462              assert shape(self) == shape(other) 
 463              if hasattr(other, 'coordnames'): 
 464                  if self.coordnames != other.coordnames: 
 465                      raise ValueError("Coordinate mismatch") 
 466              return linalg.norm(self.coordarray, self._normord) <= \
 
 467                     linalg.norm(other.coordarray, self._normord) 
 468          except (AttributeError, TypeError, AssertionError): 
 469              return self.coordarray <= other 
 470          except ZeroDivisionError: 
 471              raise ValueError("Norm order for point cannot be zero")

 472  
 


473 -    def __ge__(self, other):


474          try: 
 475              assert shape(self) == shape(other) 
 476              if hasattr(other, 'coordnames'): 
 477                  if self.coordnames != other.coordnames: 
 478                      raise ValueError("Coordinate mismatch") 
 479              return linalg.norm(self.coordarray, self._normord) >= \
 
 480                     linalg.norm(other.coordarray, self._normord) 
 481          except (AttributeError, TypeError, AssertionError): 
 482              return self.coordarray >= other 
 483          except ZeroDivisionError: 
 484              raise ValueError("Norm order for point cannot be zero")

 485  
 


486 -    def __eq__(self, other):


487          try: 
 488              assert shape(self) == shape(other) 
 489              if hasattr(other, 'coordnames'): 
 490                  if self.coordnames != other.coordnames: 
 491                      raise ValueError("Coordinate mismatch") 
 492              return linalg.norm(self.coordarray, self._normord) == \
 
 493                     linalg.norm(other.coordarray, self._normord) 
 494          except (AttributeError, TypeError, AssertionError): 
 495              return self.coordarray == other 
 496          except ZeroDivisionError: 
 497              raise ValueError("Norm order for point cannot be zero")

 498  
 


499 -    def __ne__(self, other):


500          try: 
 501              assert shape(self) == shape(other) 
 502              if hasattr(other, 'coordnames'): 
 503                  if self.coordnames != other.coordnames: 
 504                      raise ValueError("Coordinate mismatch") 
 505              return linalg.norm(self.coordarray, self._normord) != \
 
 506                     linalg.norm(other.coordarray, self._normord) 
 507          except (AttributeError, TypeError, AssertionError): 
 508              return self.coordarray != other 
 509          except ZeroDivisionError: 
 510              raise ValueError("Norm order for point cannot be zero")

 511  
 
 512  
 


513 -    def _infostr(self, verbose=0):


514          precision = 8 
 515          if verbose == 0: 
 516              outputStr = "Point with coords:\n" 
 517              for c in self.coordnames: 
 518                  outputStr += c 
 519                  if c != self.coordnames[-1]: 
 520                      outputStr += "\n" 
 521          elif verbose > 0: 
 522              outputStr = '' 
 523              for c in self.coordnames: 
 524                  v = self.coordarray[self._map_names_to_ixs(c)] 
 525                  if isinstance(v, ndarray): 
 526                      dvstr = str(v[0]) 
 527                  else: 
 528                      # only alternative is a singleton numeric value (not list)
 
 529                      dvstr = str(v) 
 530                  outputStr += c+':  '+dvstr 
 531                  if c != self.coordnames[-1]: 
 532                      outputStr += "\n" 
 533              for label, infodict in self.labels.iteritems(): 
 534                  outputStr += "\nLabels: %s (%s)"%(label, str(infodict)) 
 535          return outputStr

 536  
 
 537  
 


538 -    def __repr__(self):


539          return self._infostr(verbose=1)

 540  
 
 541  
 
 542      __str__ = __repr__ 
 543  
 
 544  
 


545 -    def info(self, verboselevel=1):


546          print self._infostr(verboselevel)

 547  
 
 548  
 


549 -    def __abs__(self):


550          return linalg.norm(self.coordarray, self._normord)

 551  
 
 552  
 


553 -    def __copy__(self):


554          return Point({'coordarray': copy(self.coordarray),
 
 555                        'coordnames': copy(self.coordnames),
 
 556                        'coordtype': self.coordtype,
 
 557                        'norm': self._normord,
 
 558                        'labels': self.labels})

 559  
 
 560      copy = __copy__ 
 561  
 
 562  
 


563 -    def __getstate__(self):


564          d = copy(self.__dict__) 
 565          # remove reference to Cfunc type
 
 566          d['coordtype'] = _num_type2name[self.coordtype] 
 567          return d

 568  
 
 569  
 


570 -    def __setstate__(self, state):


571          self.__dict__.update(state) 
 572          # reinstate Cfunc type
 
 573          self.coordtype = _num_name2type[self.coordtype]

 574  
 
 575  
 
 576  #----------------------------------------------------------------------------
 
 577  
 
 578  
 


579 -class Pointset(Point):


580      """1D parameterized or non-parameterized set of discrete points.
 
 581      (If present, the independent variable must be a float64 or an int32)""" 
 582  
 


583 -    def __init__(self, kwd=None, **kw):


584          if kwd is not None: 
 585              if kw != {}: 
 586                  raise ValueError("Cannot mix keyword dictionary and keywords") 
 587              kw = kwd 
 588              if intersect(kw.keys(), point_keys) == []: 
 589                  # creating Pointset from dictionary
 
 590                  temp_kw = {} 
 591                  temp_kw['coorddict'] = copy(kw) 
 592                  kw = copy(temp_kw) 
 593          # Deal with independent variable, if present
 
 594          if 'indepvardict' in kw: 
 595              assert len(kw['indepvardict']) == 1 
 596              try: 
 597                  it = kw['indepvartype'] 
 598              except KeyError: 
 599                  self.indepvartype = float64 
 600              else: 
 601                  try: 
 602                      self.indepvartype = _num_equivtype[it] 
 603                  except KeyError: 
 604                      raise TypeError('Independent variable type %s not valid'%str(it)) 
 605              vals = kw['indepvardict'].values()[0] 
 606              self.indepvarname = kw['indepvardict'].keys()[0] 
 607              if isinstance(vals, _seq_types): 
 608                  self.indepvararray = array(vals, self.indepvartype) 
 609              else: 
 610                  try: 
 611                      assert self.indepvartype == _num_equivtype[type(vals)] 
 612                  except (AssertionError, KeyError): 
 613                      raise TypeError("Invalid type for independent variable value") 
 614                  else: 
 615                      self.indepvararray = array([vals], self.indepvartype) 
 616          elif 'indepvararray' in kw: 
 617              if 'indepvarname' in kw: 
 618                  self.indepvarname = kw['indepvarname'] 
 619              else: 
 620                  self.indepvarname = 't' 
 621              vals = kw['indepvararray'] 
 622              if isinstance(vals, list): 
 623                  try: 
 624                      it = kw['indepvartype'] 
 625                  except: 
 626                      self.indepvartype = float64 
 627                  else: 
 628                      try: 
 629                          self.indepvartype = _num_equivtype[it] 
 630                      except KeyError: 
 631                          raise TypeError('Independent variable type %s not valid'%str(it)) 
 632                  self.indepvararray = array(vals, self.indepvartype) 
 633              elif isinstance(vals, ndarray): 
 634                  # call 'array' constructor to ensure copy is made in case
 
 635                  # either array is independently changed.
 
 636                  if rank(vals) == 0: 
 637                      self.indepvararray = array(vals.ravel()) 
 638                  else: 
 639                      self.indepvararray = array(vals) 
 640                  try: 
 641                      self.indepvartype = _num_equivtype[self.indepvararray.dtype.type] 
 642                  except KeyError: 
 643                      raise TypeError('Independent variable type '
 
 644                                      '%s not valid'%self.indepvararray.dtype) 
 645              else: 
 646                  raise TypeError("Invalid type for independent variable "
 
 647                                  "array: "+str(type(vals))) 
 648  
 
 649          else: 
 650              # non-parameterized case
 
 651              self.indepvarname = None 
 652              self.indepvartype = None 
 653              self.indepvararray = None 
 654              self._parameterized = False 
 655          if self.indepvarname: 
 656              # do validation checks
 
 657              assert isinstance(self.indepvarname, str), \
 
 658                     'independent variable name must be a string' 
 659              try: 
 660                  self.indepvartype = _num_equivtype[self.indepvararray.dtype.type] 
 661              except KeyError: 
 662                  raise TypeError('Independent variable type '
 
 663                                      '%s not valid'%self.indepvararray.dtype) 
 664              r=rank(self.indepvararray) 
 665              if r == 1: 
 666                  pass 
 667              elif r == 0: 
 668                  self.indepvararray = self.indepvararray.ravel() 
 669              else: 
 670                  raise ValueError("Invalid rank for "
 
 671                                  "independent variable array %i"%r) 
 672              # if user gave independent variable array in reverse order,
 
 673              # then we'll reverse this and the coord arrays and the labels
 
 674              # at the end of initialization
 
 675              do_reverse = not isincreasing(self.indepvararray) 
 676              self._parameterized = True 
 677          # Deal with coordinate data
 
 678          if 'coorddict' in kw: 
 679              coorddict = {} 
 680              try: 
 681                  ct = kw['coordtype'] 
 682              except KeyError: 
 683                  self.coordtype = float64 
 684              else: 
 685                  try: 
 686                      self.coordtype = _num_equivtype[ct] 
 687                  except KeyError: 
 688                      raise TypeError('Coordinate type %s not valid for Point'%str(ct)) 
 689              for c, v in kw['coorddict'].iteritems(): 
 690                  if isinstance(c, str): 
 691                      c_key = c 
 692                  else: 
 693                      c_key = repr(c) 
 694                  if isinstance(v, list): 
 695                      coorddict[c_key] = array(v, self.coordtype) 
 696                  elif isinstance(v, ndarray): 
 697                      # call 'array' constructor on array to ensure it is a copy
 
 698                      # if either array is independently changed.
 
 699                      coorddict[c_key] = array(v, self.coordtype) 
 700                  elif isinstance(v, Pointset): 
 701                      coorddict[c_key] = v.toarray() 
 702                  else: 
 703                      try: 
 704                          assert self.coordtype == _num_equivtype[type(v)] 
 705                      except (AssertionError, KeyError): 
 706                          raise TypeError("Must pass arrays, lists, or numeric types") 
 707                      else: 
 708                          coorddict[c_key] = array([v], self.coordtype) 
 709              self.coordnames = coorddict.keys() 
 710              # only way to order dictionary keys for array is to sort
 
 711              self.coordnames.sort() 
 712              self.dimension = len(self.coordnames) 
 713              datalist = [] 
 714              # loop over coordnames to ensure correct ordering of coordarray
 
 715              if self._parameterized: 
 716                  my_len = len(self.indepvararray) 
 717              else: 
 718                  my_len = len(coorddict[self.coordnames[0]]) 
 719              for c in self.coordnames: 
 720                  xs = coorddict[c] 
 721                  if my_len != len(xs): 
 722                      if self._parameterized: 
 723                          raise ValueError('Independent variable array length must match '
 
 724                             'that of each coordinate array') 
 725                      else: 
 726                          raise ValueError('All coordinate arrays must have same length') 
 727                  datalist.append(xs) 
 728              self.coordarray = array(datalist, self.coordtype) 
 729              r = rank(self.coordarray) 
 730              if r == 2: 
 731                  pass 
 732              elif r == 1: 
 733                  self.coordarray = array([self.coordarray], self.coordtype) 
 734              elif r == 0: 
 735                  self.coordarray = array([self.coordarray.ravel()], self.coordtype) 
 736              else: 
 737                  raise ValueError("Invalid rank for coordinate array: %i"%r) 
 738              assert self.dimension == self.coordarray.shape[0], "Invalid coord array" 
 739          elif 'coordarray' in kw: 
 740              if not isinstance(kw['coordarray'], _seq_types): 
 741                  raise TypeError('Coordinate type %s not valid for Pointset'%str(type(kw['coordarray']))) 
 742              try: 
 743                  ct = kw['coordtype'] 
 744              except KeyError: 
 745                  self.coordtype = float64 
 746              else: 
 747                  try: 
 748                      self.coordtype = _num_equivtype[ct] 
 749                  except KeyError: 
 750                      raise TypeError('Coordinate type %s not valid'%str(ct)) 
 751              # calling 'array' constructor creates a copy if original or new
 
 752              # array is altered
 
 753              array_temp = array(kw['coordarray'], self.coordtype) 
 754              r = rank(array_temp) 
 755              if r == 2: 
 756                  self.coordarray = array_temp 
 757              elif r == 1: 
 758                  self.coordarray = array([kw['coordarray']], self.coordtype) 
 759              elif r == 0: 
 760                  self.coordarray = array([array_temp.ravel()], self.coordtype) 
 761              else: 
 762                  raise ValueError("Invalid rank for coordinate array %i"%r) 
 763              self.dimension = self.coordarray.shape[0] 
 764              if 'coordnames' in kw: 
 765                  if isinstance(kw['coordnames'], str): 
 766                      coordnames = [kw['coordnames']] 
 767                  else: 
 768                      coordnames = kw['coordnames'] 
 769              else: 
 770                  coordnames = [str(cix) for cix in range(self.dimension)] 
 771              if len(coordnames) != self.dimension: 
 772                  print "Pointset initialization error:" 
 773                  print "Found Coordnames: ", coordnames, \
 
 774                               "(dimension = %s)"%len(coordnames) 
 775                  print "vs. data dimension =", self.dimension 
 776                  raise ValueError("Mismatch between number of coordnames and "
 
 777                                   "dimension of data") 
 778              cs = array(coordnames) 
 779              order = cs.argsort() 
 780              self.coordnames = cs[order].tolist() 
 781              self.coordarray = take(self.coordarray,order,axis=0) 
 782              self.coordtype = self.coordarray.dtype.type 
 783          else: 
 784              raise ValueError("Missing coord info in keywords") 
 785          assert isUniqueSeq(self.coordnames), 'Coordinate names must be unique' 
 786          self.makeIxMaps() 
 787          if self._parameterized: 
 788              assert self.indepvarname not in self.coordnames, \
 
 789                     "Independent variable name appeared in coordinate names" 
 790              #            if len(self.coordarray.shape) > 1:
 
 791              assert self.coordarray.shape[1] == len(self.indepvararray), \
 
 792                     ("Coord array length mismatch with independent variable"
 
 793                      " array length") 
 794              #else:
 
 795              #    assert self.coordarray.shape[0] == len(self.indepvararray)
 
 796              # process choice of indep var tolerance
 
 797              if 'checklevel' in kw: 
 798                  checklevel = kw['checklevel'] 
 799                  if checklevel in [0,1]: 
 800                      self.checklevel = checklevel 
 801                  else: 
 802                      raise ValueError("Invalid check level") 
 803              else: 
 804                  # default to use tolerance in indep val resolution
 
 805                  self.checklevel = 1 
 806              if 'tolerance' in kw: 
 807                  tol = kw['tolerance'] 
 808                  if tol > 0: 
 809                      self._abseps = tol 
 810                  else: 
 811                      raise ValueError("Tolerance must be a positive real number") 
 812              else: 
 813                  self._abseps = 1e-13 
 814          if 'name' in kw: 
 815              if isinstance(kw['name'], str): 
 816                  self.name = kw['name'] 
 817              else: 
 818                  raise TypeError("name argument must be a string") 
 819          else: 
 820              self.name = "" 
 821          if 'norm' in kw: 
 822              if kw['norm'] == 0: 
 823                  raise ValueError("Norm order for point cannot be zero") 
 824              self._normord = kw['norm'] 
 825          else: 
 826              self._normord = 2 
 827          if 'labels' in kw: 
 828              try: 
 829                  self.labels = PointInfo(kw['labels'].by_index) 
 830              except AttributeError: 
 831                  self.labels = PointInfo(kw['labels']) 
 832          else: 
 833              self.labels = PointInfo() 
 834          if 'tags' in kw: 
 835              self.tags = kw['tags'] 
 836          else: 
 837              self.tags = {} 
 838          if self._parameterized: 
 839              if do_reverse: 
 840                  # finish the operation of reversing the reverse-order
 
 841                  # input arrays
 
 842                  self.indepvararray = self.indepvararray[::-1] 
 843                  self.reverse() 
 844              if not isincreasing(self.indepvararray): 
 845                  raise ValueError("Independent variable values must be in "
 
 846                                         "increasing order")

 847  
 
 848  
 


849 -    def __delitem__(self, k):


850          """Remove point by index or by coordinate.""" 
 851          if k in self.coordnames: 
 852              cs = remain(self.coordnames, k) 
 853              p_result = copy(self[cs]) 
 854              self.coordnames = cs 
 855              self.coordarray = p_result.coordarray 
 856              self.labels = p_result.labels 
 857              self.indepvararray = p_result.indepvararray 
 858              self.makeIxMaps() 
 859          else: 
 860              # assume integer
 
 861              self.remove(k)

 862  
 
 863  
 


864 -    def remove(self, ix):


865          """Remove individual Point by its index.""" 
 866          if ix == 0: 
 867              try: 
 868                  p_result = copy(self[1:]) 
 869              except ValueError: 
 870                  # slice index out of range => only 1 point left!
 
 871                  raise ValueError("Cannot remove only point in pointset!") 
 872          else: 
 873              ix = ix % len(self) 
 874              p_result = copy(self[:ix]) 
 875              try: 
 876                  p_result.append(self[ix+1:]) 
 877              except ValueError: 
 878                  # ix was at end, so nothing left to append
 
 879                  pass 
 880          self.coordarray = p_result.coordarray 
 881          self.labels = p_result.labels 
 882          self.indepvararray = p_result.indepvararray 
 883          self.makeIxMaps()

 884  
 
 885  
 


886 -    def reverse(self):


887          """Reverse order of points *IN PLACE*.""" 
 888          self.coordarray = self.coordarray[:,::-1] 
 889          self.labels.mapIndices(dict(zip(range(0,len(self)),range(len(self)-1,-1,-1))))

 890  
 


891 -    def rename(self, coord, newcoord):


892          """Rename a coordinate.""" 
 893          try: 
 894              ix = self.coordnames.index(coord) 
 895          except ValueError: 
 896              raise ValueError("No such coordinate: %s"%coord) 
 897          self.coordnames[ix] = newcoord 
 898          self.makeIxMaps()

 899  
 


900 -    def makeIxMaps(self):


901          self._name_ix_map = dict(zip(self.coordnames, range(self.dimension))) 
 902          self._ix_name_map = copy(self.coordnames) 
 903          if self._parameterized: 
 904              self._indepvar_ix_map = makeArrayIxMap(self.indepvararray) 
 905          else: 
 906              self._indepvar_ix_map = None

 907  
 
 908  
 


909 -    def addlabel(self, ix, label, info=None):


910          """Add string label to indexed point. info dictionary is optional""" 
 911          if ix in range(len(self)): 
 912              self.labels.update(ix, label, info) 
 913          else: 
 914              raise ValueError("Index out of range")

 915  
 
 916  
 


917 -    def removelabel(self, ix):


918          """Remove all labels at indexed point.""" 
 919          del self.labels[ix]

 920  
 
 921  
 


922 -    def bylabel(self, s):


923          """Return pointset containing points labelled with the supplied
 
 924          labels. Argument s can be a string or a list of strings.""" 
 925          if isinstance(s, str): 
 926              if s == '': 
 927                  raise ValueError("Label must be non-empty") 
 928              else: 
 929                  ixlist = sortedDictKeys(self.labels[s]) 
 930                  if ixlist != []: 
 931                      return self[ixlist] 
 932                  else: 
 933                      return None 
 934          elif isinstance(s, list): 
 935              ixlist = [] 
 936              for ss in s: 
 937                  if isinstance(ss, str): 
 938                      if ss == '': 
 939                          raise ValueError("Label must be non-empty") 
 940                      ixlist = sortedDictKeys(self.labels[ss]) 
 941                  else: 
 942                      raise TypeError("Invalid label type") 
 943              if ixlist != []: 
 944                  return self[ixlist] 
 945              else: 
 946                  return None 
 947          else: 
 948              raise TypeError("Invalid label type")

 949  
 
 950  
 
 951  
 


952 -    def __setitem__(self, ix, p):


953          """Change individual points, accessed by index (no slicing supported).
 
 954          Individual coordinate values of a point can be changed by adding a
 
 955          cross-reference coordinate name or index.
 
 956          If ix is a variable name then the entire row can be changed (again,
 
 957          no slicing supported).""" 
 958          if isinstance(ix, _int_types): 
 959              if isinstance(p, Point): 
 960                  if compareNumTypes(self.coordtype, int32) and \
 
 961                     compareNumTypes(p.coordtype, float64): 
 962                      raise ValueError("Cannot update integer pointset with a float") 
 963                  self.coordarray[:,ix] = p.toarray() 
 964                  if len(p.labels) > 0: 
 965                      self.labels.update({ix: p.labels}) 
 966              elif isinstance(p, dict): 
 967                  vlist = [] 
 968                  for k in self.coordnames: 
 969                      vlist.append(p[k]) 
 970                  self.coordarray[:,ix] = array(vlist, self.coordtype) 
 971              elif isinstance(p, _seq_types): 
 972                  self.coordarray[:,ix] = array(p, self.coordtype) 
 973              else: 
 974                  raise TypeError("Invalid index reference") 
 975          elif isinstance(ix, tuple) and len(ix) == 2: 
 976              # note that index order must be reversed
 
 977              try: 
 978                  c = self._name_ix_map[ix[1]] 
 979              except KeyError: 
 980                  c = ix[1] 
 981              if isinstance(p, _int_types): 
 982                  self.coordarray[c,ix[0]] = p 
 983              elif isinstance(p, _float_types): 
 984                  if self.coordtype == float64: 
 985                      self.coordarray[c,ix[0]] = p 
 986                  else: 
 987                      raise TypeError("Cannot update an integer pointset with a float") 
 988              elif isinstance(p, ndarray) and p.shape==(1,): 
 989                  self.coordarray[c,ix[0]] = p[0] 
 990              elif isinstance(p, list) and len(list) == 1: 
 991                  self.coordarray[c,ix[0]] = p[0] 
 992              elif isinstance(p, Point) and p.dimension == 1: 
 993                  self.coordarray[c,ix[0]] = p[0] 
 994                  if len(p.labels) > 0: 
 995                      self.labels.update({ix: p.labels}) 
 996              else: 
 997                  raise TypeError("New value is not a singleton numeric type") 
 998          elif isinstance(ix, str): 
 999              if ix == self.indepvarname: 
1000                  if isinstance(p, Pointset): 
1001                      if compareNumTypes(self.indepvartype, int32) and \
 
1002                         compareNumTypes(p.indepvartype, float64): 
1003                          raise ValueError("Cannot update integer independent variable with a float") 
1004                      if len(self) == len(p): 
1005                          self.indepvararray = p.toarray() 
1006                      else: 
1007                          raise ValueError("Size mismatch for new independent variable array") 
1008                      # labels ignored
 
1009                  elif isinstance(p, dict): 
1010                      if len(self) == len(p[c]): 
1011                          self.indepvararray = array(p[c], self.indepvartype) 
1012                      else: 
1013                          raise ValueError("Size mismatch for new independent variable array") 
1014                  elif isinstance(p, _seq_types): 
1015                      if len(self) == len(p): 
1016                          self.indepvararray = array(p, self.indepvartype) 
1017                      else: 
1018                          raise ValueError("Size mismatch for new independent variable array") 
1019                  else: 
1020                      raise TypeError("Invalid data") 
1021              elif ix in self.coordnames: 
1022                  c = self._name_ix_map[ix] 
1023                  if isinstance(p, Pointset): 
1024                      if compareNumTypes(self.coordtype, int32) and \
 
1025                         compareNumTypes(p.coordtype, float64): 
1026                          raise ValueError("Cannot update integer pointset with a float") 
1027                      self.coordarray[c,:] = p.toarray() 
1028                      # labels ignored
 
1029                  elif isinstance(p, dict): 
1030                      self.coordarray[c,:] = array(p[c], self.coordtype) 
1031                  elif isinstance(p, _seq_types): 
1032                      self.coordarray[c,:] = array(p, self.coordtype) 
1033                  elif isinstance(p, _real_types): 
1034                      self.coordarray[c,:] = float(p) 
1035                  else: 
1036                      raise TypeError("Invalid data") 
1037              else: 
1038                  raise TypeError("Invalid variable reference") 
1039          else: 
1040              raise TypeError("Invalid Pointset reference")

1041  
 
1042  
 


1043 -    def __getitem__(self, ix):


1044          # select points
 
1045          if isinstance(ix, _int_types): 
1046              # The labels (PointInfo) object doesn't understand -ve indices,
 
1047              # but don't take modulo length otherwise iteration will break
 
1048              if ix < 0: 
1049                  ix = ix + self.coordarray.shape[1] 
1050              if ix in self.labels: 
1051                  label = self.labels[ix] 
1052              else: 
1053                  label = {} 
1054              return Point({'coordarray': self.coordarray[:,ix],
 
1055                            'coordnames': self.coordnames,
 
1056                            'norm': self._normord,
 
1057                            'labels': label}) 
1058          elif isinstance(ix, tuple): 
1059              if len(ix) != 2: 
1060                  raise ValueError("Only use 2-tuples in referencing pointset") 
1061              ref1 = ix[0] 
1062              ref2 = ix[1] 
1063          elif isinstance(ix, str): 
1064              # reference by coord name
 
1065              if self._parameterized: 
1066                  if ix == self.indepvarname: 
1067                      return self.indepvararray 
1068                  else: 
1069                      return self.coordarray[self._map_names_to_ixs(ix),:] 
1070              else: 
1071                  return self.coordarray[self._map_names_to_ixs(ix),:] 
1072          elif isinstance(ix, list): 
1073              if all([x in self.coordnames for x in ix]): 
1074                  ref1 = slice(len(self)) 
1075                  ref2 = ix 
1076              else: 
1077                  ref1 = ix 
1078                  ref2 = None 
1079          elif isinstance(ix, (ndarray, slice)): 
1080              ref1 = ix 
1081              ref2 = None 
1082          else: 
1083              raise IndexError("Illegal index %s"%str(ix)) 
1084          if isinstance(ref1, (list, ndarray, _int_types)): 
1085              if isinstance(ref1, _int_types): 
1086                  ref1 = [ref1] 
1087              try: 
1088                  ca = take(self.coordarray, ref1, axis=1) 
1089              except ValueError: 
1090                  raise ValueError("Invalid variable names given: "%(str(ref1))) 
1091              try: 
1092                  ci = take(self.indepvararray, ref1, axis=0) 
1093              except (IndexError, AttributeError): 
1094                  # non-parameterized pointset
 
1095                  pass 
1096              cl = self.labels[ref1] 
1097              cl_ixs = cl.getIndices() 
1098              ixmap = invertMap(ref1) 
1099              new_cl_ixs = [ixmap[i] for i in cl_ixs] 
1100          elif isinstance(ref1, slice): 
1101              ls = len(self) 
1102              if ref1.stop is None: 
1103                  stop = ls 
1104              else: 
1105                  if ref1.stop < 0: 
1106                      stop = ref1.stop + self.coordarray.shape[1] + 1 
1107                  else: 
1108                      stop = ref1.stop 
1109              s1, s2, s3 = ref1.indices(stop) 
1110              if s1 < 0 or s2 > ls or s1 >= ls: 
1111                  raise ValueError("Slice index out of range") 
1112              ca = take(self.coordarray, xrange(s1, s2, s3), axis=1) 
1113              try: 
1114                  ci = take(self.indepvararray, xrange(s1, s2, s3),axis=0) 
1115              except (IndexError, AttributeError): 
1116                  # non-parameterized pointset
 
1117                  pass 
1118              cl = self.labels[ref1] 
1119              cl_ixs = cl.getIndices() 
1120              lowest_ix = ref1.start or 0 
1121              if lowest_ix < 0: 
1122                  lowest_ix = len(self)+lowest_ix 
1123              new_cl_ixs = [i-lowest_ix for i in cl_ixs] 
1124          else: 
1125              print "ref1 argument =", ref1 
1126              raise TypeError("Type %s is invalid for Pointset indexing"%str(type(ref1))) 
1127          ixlist = self._force_coords_to_ixlist(ref2) 
1128          ca = take(ca, ixlist, axis=0) 
1129          try: 
1130              cl.mapIndices(dict(zip(cl_ixs, new_cl_ixs))) 
1131          except AttributeError: 
1132              pass 
1133          if self._parameterized: 
1134              return Pointset({'coordarray': ca,
 
1135                               'coordnames': [self.coordnames[i] for i in ixlist],
 
1136                               'indepvararray': ci,
 
1137                               'indepvarname': self.indepvarname,
 
1138                               'norm': self._normord,
 
1139                               'labels': cl}) 
1140          else: 
1141              return Pointset({'coordarray': ca,
 
1142                              'coordnames': [self.coordnames[i] for i in ixlist],
 
1143                              'norm': self._normord,
 
1144                              'labels': cl})

1145  
 
1146  
 


1147 -    def _resolve_indepvar(self, p):


1148          if self.checklevel == 0: 
1149              return self._indepvar_ix_map[p] 
1150          else: 
1151              try: 
1152                  return self._indepvar_ix_map[p] 
1153              except: 
1154                  ixs = self.findIndex(p) 
1155                  lval = self.indepvararray[ixs[0]] 
1156                  rval = self.indepvararray[ixs[1]] 
1157                  if p - lval < self._abseps: 
1158                      return ixs[0] 
1159                  elif rval - p <= self._abseps: 
1160                      return ixs[1] 
1161                  else: 
1162                      lerr = p - lval 
1163                      rerr = rval - p 
1164                      raise KeyError( \
 
1165                    "%f not found in (%f, %f) @tol=%.16f: mismatches=(%.16f, %.16f)"%(p,lval,rval,self._abseps,lerr,rerr))

1166  
 
1167  
 


1168 -    def setTol(self, tol):


1169          if tol > 0: 
1170              self._abseps = tol 
1171          else: 
1172              raise ValueError("tolerance must be a positive real number")

1173  
 
1174  
 


1175 -    def __call__(self, p, coords=None):


1176          if not self._parameterized: 
1177              raise TypeError("Cannot call a non-parameterized Pointset") 
1178          if isinstance(p, _seq_types): 
1179              # assume p is an all-numeric list, so it should be treated as
 
1180              # an independent variable.
 
1181              try: 
1182                  ix = [self._resolve_indepvar(i) for i in p] 
1183              except KeyError: 
1184                  raise ValueError("Independent variable value not valid: %s"%str(p)) 
1185          else: 
1186              # assume p is an integer or float, appropriate to independent var
 
1187              try: 
1188                  ix = self._resolve_indepvar(p) 
1189              except KeyError: 
1190                  raise ValueError("Independent variable value not valid: " \
 
1191                                   + str(p)) 
1192          if coords is None: 
1193              if isinstance(ix, _int_types): 
1194                  label = self.labels[ix] 
1195                  try: 
1196                      label.mapIndices({ix: 0}) 
1197                  except AttributeError: 
1198                      # empty
 
1199                      pass 
1200                  return Point({'coordarray': self.coordarray[:,ix],
 
1201                                'coordnames': self.coordnames,
 
1202                                'norm': self._normord,
 
1203                                'labels': label}) 
1204              else: 
1205                  labels = self.labels[ix] 
1206                  cl_ixs = labels.getIndices() 
1207                  ixmap = invertMap(ix) 
1208                  new_cl_ixs = [ixmap[i] for i in cl_ixs] 
1209                  if isinstance(ix, slice): 
1210                      lowest_ix = ix.start or 0 
1211                      new_cl_ixs = [i-lowest_ix for i in cl_ics] 
1212                  elif isinstance(ix, (list, ndarray)): 
1213                      new_cl_ixs = [ixmap[i] for i in cl_ixs] 
1214                  try: 
1215                      labels.mapIndices(dict(zip(cl_ixs, new_cl_ixs))) 
1216                  except AttributeError: 
1217                      # empty
 
1218                      pass 
1219                  return Pointset({'coordarray': take(self.coordarray, ix, axis=1),
 
1220                           'coordnames': self.coordnames,
 
1221                           'indepvarname': self.indepvarname,
 
1222                           'indepvararray': take(self.indepvararray, ix, axis=0),
 
1223                           'norm': self._normord,
 
1224                           'labels': labels}) 
1225          else: 
1226              clist = self._force_coords_to_ixlist(coords) 
1227              if isinstance(ix, _int_types): 
1228                  label = self.labels[ix] 
1229                  try: 
1230                      label.mapIndices({ix: 0}) 
1231                  except AttributeError: 
1232                      # empty
 
1233                      pass 
1234                  return Point({'coordarray': self.coordarray[clist, ix],
 
1235                            'coordnames': [self.coordnames[i] for i in clist],
 
1236                            'norm': self._normord,
 
1237                            'labels': label}) 
1238              else: 
1239                  labels = self.labels[ix] 
1240                  try: 
1241                      labels.mapIndices(dict(zip(labels, [i-ix[0] for i in labels.getIndices()]))) 
1242                  except AttributeError: 
1243                      # empty
 
1244                      pass 
1245                  return Pointset({'coordarray': take(self.coordarray[clist], ix, axis=1),
 
1246                                   'coordnames': [self.coordnames[i] for i in clist],
 
1247                                   'indepvarname': self.indepvarname,
 
1248                                   'indepvararray': take(self.indepvararray, ix, axis=0),
 
1249                                   'norm': self._normord,
 
1250                                   'labels': labels})

1251  
 
1252  
 


1253 -    def __len__(self):


1254          return self.coordarray.shape[1]

1255  
 
1256  
 


1257 -    def __contains__(self, other):


1258          for i in xrange(len(self)): 
1259              if comparePointCoords(self.__getitem__(i), other): 
1260                  return True 
1261          return False

1262  
 
1263  
 


1264 -    def __lt__(self, other):


1265          if isinstance(other, Pointset): 
1266              if not all(self.indepvararray == other.indepvararray): 
1267                  raise ValueError("Independent variable arrays are not the same") 
1268              return array([self[i] < other[i] for i in range(len(self))], 'Bool') 
1269          elif isinstance(other, Point): 
1270              return array([p < other for p in self], 'Bool') 
1271          else: 
1272              try: 
1273                  return self.coordarray < other 
1274              except: 
1275                  raise TypeError("Invalid type for comparison with Pointset")

1276  
 


1277 -    def __gt__(self, other):


1278          if isinstance(other, Pointset): 
1279              if not all(self.indepvararray == other.indepvararray): 
1280                  raise ValueError("Independent variable arrays are not the same") 
1281              return array([self[i] > other[i] for i in range(len(self))], 'Bool') 
1282          elif isinstance(other, Point): 
1283              return array([p > other for p in self], 'Bool') 
1284          else: 
1285              try: 
1286                  return self.coordarray > other 
1287              except: 
1288                  raise TypeError("Invalid type for comparison with Pointset")

1289  
 


1290 -    def __le__(self, other):


1291          if isinstance(other, Pointset): 
1292              if not all(self.indepvararray == other.indepvararray): 
1293                  raise ValueError("Independent variable arrays are not the same") 
1294              return array([self[i] <= other[i] for i in range(len(self))], 'Bool') 
1295          elif isinstance(other, Point): 
1296              return array([p <= other for p in self], 'Bool') 
1297          else: 
1298              try: 
1299                  return self.coordarray <= other 
1300              except: 
1301                  raise TypeError("Invalid type for comparison with Pointset")

1302  
 


1303 -    def __ge__(self, other):


1304          if isinstance(other, Pointset): 
1305              if not all(self.indepvararray == other.indepvararray): 
1306                  raise ValueError("Independent variable arrays are not the same") 
1307              return array([self[i] >= other[i] for i in range(len(self))], 'Bool') 
1308          elif isinstance(other, Point): 
1309              return array([p >= other for p in self], 'Bool') 
1310          else: 
1311              try: 
1312                  return self.coordarray >= other 
1313              except: 
1314                  raise TypeError("Invalid type for comparison with Pointset")

1315  
 


1316 -    def __eq__(self, other):


1317          if isinstance(other, Pointset): 
1318              if not all(self.indepvararray == other.indepvararray): 
1319                  raise ValueError("Independent variable arrays are not the same") 
1320              return array([self[i] == other[i] for i in range(len(self))], 'Bool') 
1321          elif isinstance(other, Point): 
1322              return array([p == other for p in self], 'Bool') 
1323          else: 
1324              try: 
1325                  return self.coordarray == other 
1326              except: 
1327                  raise TypeError("Invalid type for comparison with Pointset")

1328  
 


1329 -    def __ne__(self, other):


1330          if isinstance(other, Pointset): 
1331              if not all(self.indepvararray == other.indepvararray): 
1332                  raise ValueError("Independent variable arrays are not the same") 
1333              return array([self[i] != other[i] for i in range(len(self))], 'Bool') 
1334          elif isinstance(other, Point): 
1335              return array([p != other for p in self], 'Bool') 
1336          else: 
1337              try: 
1338                  return self.coordarray != other 
1339              except: 
1340                  raise TypeError("Invalid type for comparison with Pointset")

1341  
 
1342  
 


1343 -    def insert(self, parg, ix=None):


1344          """Insert individual Point or Pointset before the given index.
 
1345  
 
1346          If ix is not given then the source and target Pointsets must
 
1347          be parameterized. In this case the Point or Pointset will be
 
1348          inserted according to the ordering of independent variable
 
1349          values.""" 
1350          p=copy(parg) 
1351          if ix is None: 
1352              if self._parameterized: 
1353                  if isinstance(p, Point) and self.indepvarname in p.coordnames: 
1354                      t = p[self.indepvarname] 
1355                      tix = self.find(t) 
1356                      if isinstance(tix, tuple): 
1357                          self.insert(p, tix[1]) 
1358                      else: 
1359                          # tix was an integer, meaning that t is
 
1360                          # already present in Pointset
 
1361                          raise ValueError("Point at independent variable"
 
1362                                           "value %f already present"%t) 
1363                  elif isinstance(p, Pointset) and p._parameterized and \
 
1364                         p.indepvarname == self.indepvarname: 
1365                      # Don't do a straight self.insert call in case the
 
1366                      # new indep var values need to be interleaved with
 
1367                      # the present ones.
 
1368                      #
 
1369                      # convert self.indepvararray and self.coordarray into lists (by self.todict())
 
1370                      iva = self.indepvararray.tolist() 
1371                      vd = self.todict(aslist=True) 
1372                      # get list of findIndex results for each of p indepvar vals
 
1373                      # add i for each one because each previous one will have been inserted,
 
1374                      # increasing the length of self.
 
1375                      if len(intersect(self._ix_name_map, p._ix_name_map)) != self.dimension: 
1376                          raise ValueError("Dimension mismatch with inserted Pointset") 
1377                      iva_p = p.indepvararray 
1378                      lenp = len(p) 
1379                      vd_p = p.todict() 
1380                      try: 
1381                          s_ixs = [self.findIndex(iva_p[i])[1]+i for i in xrange(lenp)] 
1382                      except TypeError: 
1383                          raise ValueError("Independent variable "
 
1384                                           "values in Pointset already present") 
1385                      p_label_ixs = p.labels.getIndices() 
1386                      s_label_ixs = self.labels.getIndices() 
1387                      sLabelMap = {} 
1388                      pLabelMap = {} 
1389                      for i in xrange(lenp): 
1390                          s_ix = s_ixs[i] 
1391                          if i in p_label_ixs: 
1392                              pLabelMap[i] = s_ix 
1393                          for s_label_ix in s_label_ixs: 
1394                              if s_label_ix >= s_ix-i: 
1395                                  sLabelMap[s_label_ix] = s_label_ix+i+1 
1396                      # for each one, list-insert new point data
 
1397                      for p_ix in xrange(lenp): 
1398                          s_ix = s_ixs[p_ix] 
1399                          iva.insert(s_ix, iva_p[p_ix]) 
1400                          for k in self._ix_name_map: 
1401                              vd[k].insert(s_ix, vd_p[k][p_ix]) 
1402                      # restore self's arrays
 
1403                      self.indepvararray = array(iva) 
1404                      datalist = [] 
1405                      for c in p._ix_name_map: 
1406                          datalist.append(vd[c]) 
1407                      self.coordarray = array(datalist, self.coordtype) 
1408                      # update labels
 
1409                      self.labels.mapIndices(sLabelMap) 
1410                      p_labels = copy(p.labels) 
1411                      p_labels.mapIndices(pLabelMap) 
1412                      self.labels.update(p_labels) 
1413                  else: 
1414                      raise TypeError("Inserted Point/Pointset must be "
 
1415                                      "parameterized and share same independent"
 
1416                                      "parameter name") 
1417              else: 
1418                  raise TypeError("Source Pointset must be parameterized") 
1419          else: 
1420              if ix > 0: 
1421                  p_result = copy(self[:ix]) 
1422                  p_result.append(p) 
1423              else: 
1424                  p_result = pointsToPointset(p, self.indepvarname) 
1425              try: 
1426                  p_result.append(self[ix:]) 
1427              except ValueError: 
1428                  # ix > greatest index, so no points left to add
 
1429                  # (i.e., p was appended to end)
 
1430                  pass 
1431              self.coordarray = p_result.coordarray 
1432              self.labels = p_result.labels 
1433              self.indepvararray = p_result.indepvararray 
1434          self.makeIxMaps()

1435  
 
1436  
 


1437 -    def append(self, parg, t=None, skipMatchingIndepvar=False):


1438          """Append individual Point or Pointset in place.
 
1439  
 
1440          skipMatchingIndepvar option causes a matching independent
 
1441          variable value at the beginning of p to be skipped (only
 
1442          meaningful for appending parameterized Pointsets). This
 
1443          option is mainly for internal use.""" 
1444  
 
1445          # test isinstance for Pointset first because it is a sub-class of Point
 
1446          # and so isinstance(p, Point) will also catch Pointsets!
 
1447          p = copy(parg) 
1448          if isinstance(p, Pointset): 
1449              assert p._parameterized == self._parameterized, "Parameterization mismatch" 
1450              # check p dimension and coordnames and type
 
1451              if compareNumTypes(self.coordtype, int32) and \
 
1452                 compareNumTypes(p.coordtype, float64): 
1453                  raise TypeError("Cannot add float64 pointset to an int32 Pointset") 
1454              pdim = p.dimension 
1455              if self._parameterized: 
1456                  if t is None: 
1457                      if self.indepvarname in p.coordnames: 
1458                          t = p[self.indepvarname] 
1459                          pdim = pdim - 1 
1460                      elif self.indepvarname == p.indepvarname: 
1461                          t = p.indepvararray 
1462                      else: 
1463                          raise ValueError("Independent variable missing from Pointset") 
1464                      if t[0] == self.indepvararray[-1] and skipMatchingIndepvar: 
1465                          tval = t[1:] 
1466                          start_ix = 1 
1467                      else: 
1468                          tval = t 
1469                          start_ix = 0 
1470                      if len(tval) > 0 and tval[0] <= self.indepvararray[-1]: 
1471                          #print tval[0], " <= ", self.indepvararray[-1]
 
1472                          raise ValueError("Independent variable value too small to add pointset") 
1473                      added_len = len(tval) 
1474                  else: 
1475                      if t[0] == self.indepvararray[-1] and skipMatchingIndepvar: 
1476                          tval = t[1:] 
1477                          start_ix = 1 
1478                      else: 
1479                          tval = t[:]  # ensures tval is an array (t might be a Pointset) 
1480                          start_ix = 0 
1481                      if len(tval) > 0 and tval[0] <= self.indepvararray[-1]: 
1482                          #print tval[0], " <= ", self.indepvararray[-1]
 
1483                          raise ValueError("Independent variable value too small to add pointset") 
1484                      added_len = len(tval) 
1485              else: 
1486                  if t is not None: 
1487                      raise TypeError("t argument cannot be used for non-parameterized pointsets") 
1488                  added_len = p.coordarray.shape[1] 
1489                  start_ix = 0 
1490              assert pdim == self.dimension, "Dimension mismatch with Pointset" 
1491              if pdim < p.dimension: 
1492                  pcoords = copy(p.coordnames) 
1493                  pcoords.remove(p.indepvarname) 
1494              else: 
1495                  pcoords = p.coordnames 
1496              if remain(pcoords, self.coordnames) != []: 
1497                  raise ValueError("Coordinate name mismatch with Pointset") 
1498              old_len = self.coordarray.shape[1] 
1499              new_len = old_len + added_len 
1500              old_coords = self.coordarray 
1501              self.coordarray = zeros((self.dimension, new_len),
 
1502                                      self.coordarray.dtype) 
1503              if self._parameterized: 
1504                  self.indepvararray.resize(new_len) 
1505                  tvals = tval[range(added_len)] 
1506                  self.indepvararray[old_len:] = tvals 
1507              for tix in xrange(old_len): 
1508                  self.coordarray[:, tix] = old_coords[:, tix] 
1509              pdict = p.todict() 
1510              self.coordarray[:, old_len:] = r_[[pdict[c][start_ix:] for c in self._ix_name_map]] 
1511              p_labels = copy(p.labels) 
1512              pixs = p.labels.getIndices() 
1513              if start_ix == 1: 
1514                  p_labels.mapIndices(dict(zip(pixs, [i+old_len-1 for i in pixs]))) 
1515              else: 
1516                  p_labels.mapIndices(dict(zip(pixs, [i+old_len for i in pixs]))) 
1517              self.labels.update(p_labels) 
1518          elif isinstance(p, Point): 
1519              # check p dimension and coordnames and type
 
1520              if compareNumTypes(self.coordtype, int32) and \
 
1521                 compareNumTypes(p.coordtype, float64): 
1522                  raise TypeError("Cannot add float64 Point to an int32 Pointset") 
1523              pdim = p.dimension 
1524              if self._parameterized: 
1525                  if t is None: 
1526                      if self.indepvarname not in p.coordnames: 
1527                          raise ValueError("Independent variable missing from Point") 
1528                      else: 
1529                          tval = p[self.indepvarname] 
1530                          if tval <= self.indepvararray[-1]: 
1531                              raise ValueError("Independent variable value too small to add Point") 
1532                          pdim = pdim - 1 
1533                  else: 
1534                      if t <= self.indepvararray[-1]: 
1535                          raise ValueError("Independent variable value too small to add Point") 
1536                      tval = t 
1537              elif t is not None: 
1538                  raise TypeError("t argument cannot be used for non-parameterized Pointsets") 
1539              assert pdim == self.dimension, "Dimension mismatch with Point" 
1540              if pdim < p.dimension: 
1541                  pcoords = copy(p.coordnames) 
1542                  if self._parameterized: 
1543                      pcoords.remove(self.indepvarname) 
1544              else: 
1545                  pcoords = p.coordnames 
1546              if remain(pcoords, self.coordnames) != []: 
1547                  raise ValueError("Coordinate name mismatch with Point") 
1548              new_len = self.coordarray.shape[1]+1 
1549              old_coords = self.coordarray 
1550              self.coordarray = zeros((self.dimension, new_len), self.coordarray.dtype) 
1551              if self._parameterized: 
1552                  self.indepvararray.resize(new_len) 
1553                  self.indepvararray.resize(new_len) 
1554                  self.indepvararray[new_len-1] = tval 
1555              for tix in xrange(new_len-1): 
1556                  self.coordarray[:, tix] = old_coords[:, tix] 
1557              for ix in xrange(self.dimension): 
1558                  self.coordarray[ix,new_len-1] = p(self._ix_name_map[ix]) 
1559              if len(p.labels) > 0: 
1560                  self.labels.update({new_len-1: p.labels}) 
1561          else: 
1562              raise TypeError("append requires Point or Pointset argument") 
1563          self.makeIxMaps()

1564  
 
1565  
 
1566      extend = append   # for intuitive compatibility! 
1567  
 
1568  
 


1569 -    def toarray(self, include_indepvar=False):


1570          """Convert the pointset to a D x L array (one variable per row),
 
1571          where D is the dimension of the pointset and L is its length.
 
1572          (This is a copy of the internal attribute 'coordarray'.)
 
1573  
 
1574          If the optional include_indepvar switch is set True (default False),
 
1575          the first row is the independent variable, and the whole
 
1576          array will be (D+1) x L in shape.
 
1577          """ 
1578          if self.dimension==1: 
1579              ca = copy(self.coordarray[0]) 
1580          else: 
1581              ca = copy(self.coordarray) 
1582          if include_indepvar: 
1583              ia = copy(self.indepvararray) 
1584              ia.shape = (1, len(ia)) 
1585              return concatenate((ia,ca)) 
1586          else: 
1587              return ca

1588  
 
1589  
 


1590 -    def todict(self, aslist=False):


1591          """Convert Pointset to a dictionary of arrays (or of lists with aslist=True).""" 
1592          if aslist: 
1593              d = dict(zip(self._ix_name_map, self.coordarray.tolist())) 
1594          else: 
1595              d = dict(zip(self._ix_name_map, self.coordarray)) 
1596              if self._parameterized: 
1597                  d[self.indepvarname] = self.indepvararray 
1598          return d

1599  
 
1600  
 


1601 -    def _infostr(self, verbose=0):


1602          if self.name == '': 
1603              outputStr = "Pointset <no name>" 
1604          else: 
1605              outputStr = "Pointset " + self.name 
1606          if self._parameterized: 
1607              outputStr += " (parameterized)" 
1608          else: 
1609              outputStr += " (non-parameterized)" 
1610          if verbose > 0: 
1611              precision = 8 
1612              lenv = len(self) 
1613              if lenv > 8: 
1614                  ixslo = range(0,2) 
1615                  ixshi = range(lenv-2,lenv) 
1616              outputStr += "\n" 
1617              if self._parameterized: 
1618                  iv = self.indepvararray 
1619                  if not isinstance(iv, ndarray): 
1620                      iv = array(iv, self.indepvartype)  # permits slicing (lists don't) 
1621                  if lenv > 8: 
1622                      alo = array2string(iv[ixslo],precision=precision) 
1623                      ahi = array2string(iv[ixshi],precision=precision) 
1624                      ivstr = alo[:-1] + ", ..., " + ahi[1:] 
1625                  else: 
1626                      ivstr = array2string(iv,precision=precision) 
1627                  outputStr += "Independent variable:\n" 
1628                  outputStr += self.indepvarname + ':  '+ivstr+"\n" 
1629              outputStr += "Coordinates:\n" 
1630              for c in self.coordnames: 
1631                  v = self.coordarray[self._map_names_to_ixs(c)] 
1632                  if not isinstance(v, ndarray): 
1633                      # only alternative is a singleton numeric value (not a list)
 
1634                      v = array([v], self.coordtype) 
1635                  if lenv > 8: 
1636                      alo = array2string(v[ixslo],precision=precision) 
1637                      ahi = array2string(v[ixshi],precision=precision) 
1638                      dvstr = alo[:-1] + ", ..., " + ahi[1:] 
1639                  else: 
1640                      dvstr = array2string(v, precision=precision) 
1641                  outputStr += c+':  '+dvstr 
1642                  if c != self.coordnames[-1]: 
1643                      outputStr += "\n" 
1644              outputStr += "\nLabels by index: " + self.labels._infostr(17) 
1645          return outputStr

1646  
 
1647  
 


1648 -    def __repr__(self):


1649          return self._infostr(verbose=1)

1650  
 
1651  
 


1652 -    def __str__(self):


1653          return self._infostr(verbose=0)

1654  
 
1655  
 


1656 -    def info(self, verboselevel=1):


1657          print self._infostr(verboselevel)

1658  
 
1659  
 


1660 -    def __copy__(self):


1661          if self._parameterized: 
1662              return Pointset({'coordarray': copy(self.coordarray),
 
1663                           'coordnames': copy(self.coordnames),
 
1664                           'indepvarname': copy(self.indepvarname),
 
1665                           'indepvararray': copy(self.indepvararray),
 
1666                           'norm': self._normord,
 
1667                           'labels': copy(self.labels)
 
1668                           }) 
1669          else: 
1670              return Pointset({'coordarray': copy(self.coordarray),
 
1671                           'coordnames': copy(self.coordnames),
 
1672                           'norm': self._normord,
 
1673                           'labels': copy(self.labels)})

1674  
 
1675      copy = __copy__ 
1676  
 
1677  
 


1678 -    def __getstate__(self):


1679          d = copy(self.__dict__) 
1680          # remove reference to Cfunc types by converting them to strings
 
1681          try: 
1682              d['indepvartype'] = _num_type2name[self.indepvartype] 
1683          except KeyError: 
1684              # non-parameterized Pointset
 
1685              pass 
1686          d['coordtype'] = _num_type2name[self.coordtype] 
1687          return d

1688  
 
1689  
 


1690 -    def __setstate__(self, state):


1691          self.__dict__.update(state) 
1692          # reinstate Cfunc types
 
1693          try: 
1694              self.indepvartype = _num_name2type[self.indepvartype] 
1695          except KeyError: 
1696              # non-parameterized Pointset
 
1697              pass 
1698          self.coordtype = _num_name2type[self.coordtype]

1699  
 


1700 -    def _match_indepvararray(self, other):


1701          """Verifies the matching of independent variable arrays in two pointsets.
 
1702          Does nothing if either object is not a parameterized pointset.""" 
1703          try: 
1704              if other._parameterized and self._parameterized: 
1705                  if not all(self.indepvararray == other.indepvararray): 
1706                      print self.indepvararray 
1707                      print other.indepvararray 
1708                      raise ValueError("Mismatched independent variable arrays") 
1709          except AttributeError: 
1710              pass

1711  
 


1712 -    def __add__(self, other):


1713          self._match_indepvararray(other) 
1714          return Point.__add__(self, other)

1715  
 


1716 -    def __radd__(self, other):


1717          self._match_indepvararray(other) 
1718          return Point.__radd__(self, other)

1719  
 


1720 -    def __sub__(self, other):


1721          self._match_indepvararray(other) 
1722          return Point.__sub__(self, other)

1723  
 


1724 -    def __rsub__(self, other):


1725          self._match_indepvararray(other) 
1726          return Point.__rsub__(self, other)

1727  
 


1728 -    def __mul__(self, other):


1729          self._match_indepvararray(other) 
1730          return Point.__mul__(self, other)

1731  
 


1732 -    def __rmul__(self, other):


1733          self._match_indepvararray(other) 
1734          return Point.__rmul__(self, other)

1735  
 


1736 -    def __div__(self, other):


1737          self._match_indepvararray(other) 
1738          return Point.__div__(self, other)

1739  
 


1740 -    def __rdiv__(self, other):


1741          self._match_indepvararray(other) 
1742          return Point.__rdiv__(self, other)

1743  
 


1744 -    def find(self, indepval, end=None):


1745          """find returns an integer index for where to place
 
1746          a point having independent variable value <indepval> in
 
1747          the Pointset, if <indepval> already exists. Otherwise, a
 
1748          pair indicating the nearest independent variable values
 
1749          present in the Pointset is returned.
 
1750  
 
1751          To ensure an integer is always returned, choose a left or
 
1752          right side to choose from the pair, using end=0 or 1 respectively.""" 
1753          if not self._parameterized: 
1754              raise TypeError("Cannot find index from independent variable for "
 
1755                              "a non-parameterized Pointset") 
1756          try: 
1757              ix = self.indepvararray.tolist().index(indepval) 
1758              result = ix 
1759          except ValueError: 
1760              cond = less(self.indepvararray, indepval).tolist() 
1761              try: 
1762                  ix = cond.index(0) 
1763                  result = (ix-1, ix) 
1764              except ValueError: 
1765                  result = (len(self.indepvararray)-1, len(self.indepvararray)) 
1766              if end is not None: 
1767                  result = result[end] 
1768          return result

1769  
 
1770      # deprecated
 
1771      findIndex = find

1772  
 
1773  
 
1774  # ----------------------------------------------------------------------------
 
1775  
 
1776  
 


1777 -class PointInfo(object):


1778      """Structure for storing individual point labels and information
 
1779      dictionaries within a Pointset object.
 
1780  
 
1781      This class will not know the size of the Pointset it is associated with,
 
1782      so index upper limits will not be checked in advance.
 
1783  
 
1784      Do not use a PointInfo object as an iterator, as it is 'infinite' in size!
 
1785      (It uses DefaultDicts as its internal storage, which return {} for
 
1786      undefined labels.)""" 
1787  
 


1788 -    def __init__(self, ptlabels=None):


1789          if ptlabels is None: 
1790              self.by_label = DefaultDict({}) 
1791              self.by_index = DefaultDict({}) 
1792          elif isinstance(ptlabels, PointInfo): 
1793              self.by_label = ptlabels.by_label 
1794              self.by_index = ptlabels.by_index 
1795          elif isinstance(ptlabels, dict): 
1796              # always expect the dictionary to be based on index
 
1797              self.by_label = DefaultDict({}) 
1798              self.by_index = DefaultDict({}) 
1799              for k, v in ptlabels.iteritems(): 
1800                  if not isinstance(k, _int_types): 
1801                      raise TypeError("Initialization dictionary must be keyed "
 
1802                                      "by integer indices") 
1803                  if isinstance(v, str): 
1804                      self.by_label[v][k] = {} 
1805                      self.by_index[k][v] = {} 
1806                  else: 
1807                      for label, infodict in v.iteritems(): 
1808                          self.by_label[label][k] = infodict 
1809                          self.by_index[k][label] = infodict 
1810          else: 
1811              raise TypeError("Invalid labels at initialization of PointInfo")

1812  
 
1813  
 


1814 -    def mapIndices(self, ixMapDict):


1815          by_index = {} 
1816          ixMap = symbolMapClass(ixMapDict) 
1817          for ix, rest in self.by_index.iteritems(): 
1818              by_index[ixMap(ix)] = rest 
1819          self.__init__(by_index)

1820  
 
1821  
 


1822 -    def mapNames(self, themap):


1823          """Map labels, using a symbol map of class symbolMapClass.""" 
1824          self.by_label = mapNames(themap, self.by_label) 
1825          new_by_index = {} 
1826          for ix, labdict in self.by_index.iteritems(): 
1827              new_by_index[ix] = mapNames(themap, labdict) 
1828          self.by_index = new_by_index

1829  
 
1830  
 


1831 -    def sortByIndex(self):


1832          ixkeys = sortedDictKeys(self.by_index) 
1833          return zip(ixkeys,[self.by_index[ix] for ix in ixkeys])

1834  
 
1835  
 


1836 -    def sortByLabel(self):


1837          labelkeys = sortedDictKeys(self.by_label) 
1838          return zip(labelkeys,[self.by_label[label] for label in labelkeys])

1839  
 
1840  
 


1841 -    def getIndices(self):


1842          return sortedDictKeys(self.by_index)

1843  
 
1844  
 


1845 -    def getLabels(self):


1846          return sortedDictKeys(self.by_label)

1847  
 
1848  
 


1849 -    def __contains__(self, key):


1850          return key in self.by_index or key in self.by_label

1851  
 
1852  
 


1853 -    def __getitem__(self, key):


1854          # indices already are enforced to be integers, and labels strings,
 
1855          # so this is a safe way to search!
 
1856          # Note: if don't use if-then test then DefaultDict will
 
1857          # create an empty entry for the failed key when .values() is called!
 
1858          if isinstance(key, tuple): 
1859              raise TypeError("Can only reference PointInfo with a single key") 
1860          else: 
1861              if isinstance(key, (slice, list, ndarray)): 
1862                  if isinstance(key, slice): 
1863                      self_ixs = self.getIndices() 
1864                      if len(self_ixs) == 0: 
1865                          max_ixs = 0 
1866                      else: 
1867                          max_ixs = max(self_ixs) 
1868                      stop = key.stop or max_ixs+1 
1869                      try: 
1870                          s1, s2, s3 = key.indices(stop) 
1871                          ixs = xrange(s1, s2, s3) 
1872                          key = intersect(ixs, self_ixs) 
1873                      except TypeError: 
1874                          key = self_ixs 
1875                  else: 
1876                      if all([isinstance(k, str) for k in key]): 
1877                          keylabels = intersect(key, self.getLabels()) 
1878                          key = [] 
1879                          for l in keylabels: 
1880                              key.extend(self.by_label[l].keys()) 
1881                          key = makeSeqUnique(key) 
1882                      elif all([isinstance(k, _int_types) for k in key]): 
1883                          key = intersect(key, self.getIndices()) 
1884                      else: 
1885                          raise TypeError("Invalid key type for PointInfo") 
1886                  return PointInfo(dict(zip(key,[self.by_index[i] for i in key]))) 
1887              elif key in self.by_index: 
1888                  return self.by_index[key] 
1889              elif key in self.by_label: 
1890                  return self.by_label[key] 
1891              elif isinstance(key, int) and key < 0: 
1892                  raise IndexError("Cannot use negative indices for PointInfo") 
1893              else: 
1894                  return {}

1895  
 
1896  
 


1897 -    def __setitem__(self, key1, the_rest):


1898          if isinstance(the_rest, tuple) and len(the_rest) == 2: 
1899              if isinstance(the_rest[0], str): 
1900                  label = the_rest[0] 
1901                  ix = None 
1902              elif isinstance(the_rest[0], _int_types): 
1903                  ix = the_rest[0] 
1904                  label = None 
1905              else: 
1906                  raise TypeError("String expected for label") 
1907              if isinstance(the_rest[1], dict): 
1908                  info = copy(the_rest[1]) 
1909              else: 
1910                  raise TypeError("Dictionary expected for info") 
1911          elif isinstance(the_rest, str): 
1912              label = the_rest 
1913              ix = None 
1914              info = {} 
1915          elif isinstance(the_rest, _int_types): 
1916              ix = the_rest 
1917              label = None 
1918              info = {} 
1919          elif isinstance(the_rest, list): 
1920              self.__setitem__(key1, the_rest[0]) 
1921              for item in the_rest[1:]: 
1922                  if isinstance(item, tuple) and len(item) == 2: 
1923                      self.update(key1, item[0], item[1]) 
1924                  else: 
1925                      self.update(key1, item) 
1926              return 
1927          else: 
1928              raise TypeError("Invalid item to set in PointInfo") 
1929          if isinstance(key1, _int_types): 
1930              if label is None: 
1931                  raise TypeError("Label expected") 
1932              ix = key1 
1933          elif isinstance(key1, str): 
1934              if ix is None: 
1935                  raise TypeError("Index expected") 
1936              label = key1 
1937          if ix < 0: 
1938              raise IndexError("Index must be non-negative") 
1939          try: 
1940              self.by_label[label].update({ix: info}) 
1941          except KeyError: 
1942              self.by_label[label] = {ix: info} 
1943          try: 
1944              self.by_index[ix].update({label: info}) 
1945          except KeyError: 
1946              self.by_index[ix] = {label: info}

1947  
 
1948  
 


1949 -    def __len__(self):


1950          return len(self.by_index)

1951  
 
1952  
 


1953 -    def remove(self, key1, *key2):


1954          """remove one or more items, keyed either by index or label.""" 
1955          byix = key1 in self.by_index 
1956          if key2 == (): 
1957              # remove all labels associated with index, or vice versa
 
1958              if byix: 
1959                  key2 = self.by_index[key1].keys() 
1960              else: 
1961                  key2 = self.by_label[key1].keys() 
1962          if byix: 
1963              for k in key2: 
1964                  # have to check k in dict otherwise DefaultDict creates entry!
 
1965                  if k in self.by_label: 
1966                      del self.by_index[key1][k] 
1967                      del self.by_label[k][key1] 
1968                  else: 
1969                      raise KeyError("Label not found") 
1970                  if self.by_label[k] == {}: 
1971                      del self.by_label[k] 
1972              if self.by_index[key1] == {}: 
1973                  del self.by_index[key1] 
1974          else: 
1975              for k in key2: 
1976                  # have to check k in dict otherwise DefaultDict creates entry!
 
1977                  if k in self.by_index: 
1978                      del self.by_index[k][key1] 
1979                      del self.by_label[key1][k] 
1980                  else: 
1981                      raise KeyError("Index not found") 
1982                  if self.by_index[k] == {}: 
1983                      del self.by_index[k] 
1984              if self.by_label[key1] == {}: 
1985                  del self.by_label[key1]

1986  
 
1987  
 


1988 -    def update(self, key1, key2=None, info=None):


1989          if isinstance(key1, PointInfo): 
1990              if key2 is None and info is None: 
1991                  for k, v in key1.by_index.iteritems(): 
1992                      for vk, vv in v.iteritems(): 
1993                          self.update(k, vk, vv) 
1994              else: 
1995                  raise TypeError("Invalid calling sequence to update") 
1996          elif isinstance(key1, dict): 
1997              if key2 is None and info is None: 
1998                  for k, v in key1.iteritems(): 
1999                      if isinstance(k, _int_types) and k >= 0: 
2000                          if isinstance(v, str): 
2001                              k2 = v 
2002                              k3 = {} 
2003                              self.update(k, k2, k3) 
2004                          elif isinstance(v, tuple) and len(v)==2: 
2005                              k2 = v[0] 
2006                              k3 = v[1] 
2007                              self.update(k, k2, k3) 
2008                          elif isinstance(v, dict): 
2009                              for k2, k3 in v.iteritems(): 
2010                                  self.update(k, k2, k3) 
2011                          else: 
2012                              raise ValueError("Invalid data for update") 
2013                      else: 
2014                          raise TypeError("Invalid index for label") 
2015              else: 
2016                  raise TypeError("Invalid calling sequence to update") 
2017          elif isinstance(key1, _int_types): 
2018              if info is None: 
2019                  info = {} 
2020              if key1 in self.by_index: 
2021                  if key2 in self.by_index[key1]: 
2022                      self.by_index[key1][key2].update(info) 
2023                  else: 
2024                      self.__setitem__(key1, (key2, info)) 
2025              else: 
2026                  self.__setitem__(key1, (key2, info)) 
2027          elif isinstance(key1, str): 
2028              if info is None: 
2029                  info = {} 
2030              if key1 in self.by_label: 
2031                  if key2 in self.by_label[key1]: 
2032                      self.by_label[key1][key2].update(info) 
2033                  else: 
2034                      self.__setitem__(key2, (key1, info)) 
2035              else: 
2036                  self.__setitem__(key2, (key1, info)) 
2037          else: 
2038              raise TypeError("Invalid type for update")

2039  
 
2040  
 


2041 -    def __delitem__(self, key):


2042          if key in self.by_index: 
2043              labels = self.by_index[key].keys() 
2044              del self.by_index[key] 
2045              for label in labels: 
2046                  del self.by_label[label][key] 
2047                  if self.by_label[label] == {}: 
2048                      del self.by_label[label] 
2049          elif key in self.by_label: 
2050              ixs = self.by_label[key].keys() 
2051              del self.by_label[key] 
2052              for ix in ixs: 
2053                  del self.by_index[ix][key] 
2054                  if self.by_index[ix] == {}: 
2055                      del self.by_index[ix] 
2056          else: 
2057              raise KeyError("Index or label not found")

2058  
 
2059  
 


2060 -    def __eq__(self, other):


2061          try: 
2062              return all(self.by_index.keys() == other.by_index.keys()) and \
 
2063                     all(self.by_label.keys() == other.by_label.keys()) 
2064          except AttributeError: 
2065              raise TypeError("Invalid type for comparison to PointInfo")

2066  
 
2067  
 


2068 -    def __ne__(self, other):


2069          return not self.__eq__(other)

2070  
 


2071 -    def _infostr(self, tab=0):


2072          lenself = len(self) 
2073          tabstr = " "*tab 
2074          basestr = ",\n"+tabstr 
2075          if lenself > 0: 
2076              entries = self.sortByIndex() 
2077              if lenself > 8: 
2078                  return basestr.join([_pretty_print_label(i) for i in entries[0:3]]) + ",\n" +\
 
2079                         (tabstr + " .\n")*3 + tabstr +\
 
2080                         basestr.join([_pretty_print_label(i) for i in entries[-3:]]) 
2081              else: 
2082                  return basestr.join([_pretty_print_label(i) for i in entries]) 
2083          else: 
2084              return "Empty"

2085  
 
2086  
 


2087 -    def __repr__(self):


2088          return self._infostr()

2089  
 
2090      __str__ = __repr__

2091  
 
2092  
 


2093 -def _pretty_print_label(d):


2094      """Internal utility to pretty print point label info.""" 
2095      s = " %s: "%repr(d[0]) 
2096      entry_keys = d[1].keys() 
2097      ki = 0 
2098      kimax = len(entry_keys) 
2099      for k in entry_keys: 
2100          keys = d[1][k].keys() 
2101          if len(keys) == 0: 
2102              s += "{%s: {}}"%k 
2103          else: 
2104              s += "{%s: {keys=%s}}"%(k,",".join(keys)) 
2105          if ki < kimax-1: 
2106              s += ', ' 
2107          ki += 1 
2108      return s

2109  
 
2110  # ------------------------------------------------
 
2111  
 
2112  
 


2113 -def comparePointCoords(p1, p2, fussy=False):


2114      """Compare two Points, Pointsets, or dictionary of point data, coordinate-wise.
 
2115      If p1 or p2 are Pointsets, their independent variable values, if present, are
 
2116      *not* compared.
 
2117  
 
2118      fussy option causes point norm order and coordinate types to be
 
2119      checked too (requires both arguments to be Points or Pointsets).
 
2120      """ 
2121      try: 
2122          p1d = dict(p1) 
2123          p1dk = p1d.keys() 
2124          p2d = dict(p2) 
2125          p2dk = p2d.keys() 
2126      except: 
2127          raise TypeError("Invalid Points, Pointsets, or dictionaries passed "
 
2128                          "to comparePointCoords") 
2129      test1 = alltrue([ks[0]==ks[1] for ks in zip(p1dk, p2dk)]) 
2130      test2 = alltrue([vs[0]==vs[1] for vs in \
 
2131                   zip([p1d[k] for k in p1dk], [p2d[k] for k in p2dk])]) 
2132      if fussy: 
2133          try: 
2134              test3 = p1._normord == p2._normord 
2135              test4 = compareNumTypes(p1.coordtype, p2.coordtype) 
2136              return test1 and test2 and test3 and test4 
2137          except AttributeError: 
2138              raise TypeError("Invalid Points, Pointsets, or dictionaries passed "
 
2139                              "to comparePointCoords with fussy option") 
2140      else: 
2141          return test1 and test2

2142  
 
2143  
 


2144 -def isparameterized(p):


2145      """Returns True if Point or Pointset p is parameterized, False otherwise""" 
2146      return p._parameterized

2147  
 
2148  
 


2149 -def makeNonParameterized(p):


2150      """Return a new Pointset stripped of its parameterization.
 
2151      """ 
2152      if isinstance(p, Pointset) and p._isparameterized: 
2153          return Pointset({'coordarray': copy(p.coordarray),
 
2154                           'coordnames': copy(p.coordnames),
 
2155                           'norm': p._normord,
 
2156                           'labels': copy(p.labels)}) 
2157      else: 
2158          raise TypeError("Must provide a parameterized Pointset")

2159  
 
2160  
 


2161 -def pointsToPointset(pointlist, indepvarname='', indepvararray=None,
 
2162                       indepvartype=float, norm=2):


2163      """Generate a Pointset from a list of Point objects (or a singleton Point).
 
2164  
 
2165      Include a name for the independent variable if constructing a
 
2166      parameterized pointset. The independent variable should be a
 
2167      coordinate of the Points passed, otherwise it can be passed as the
 
2168      optional third argument.
 
2169      """ 
2170  
 
2171      if not isinstance(indepvarname, str): 
2172          raise TypeError("String expected for independent variable name") 
2173      if isinstance(pointlist, Point): 
2174          pointlist = [pointlist] 
2175      coordnames = [] 
2176      ptype = '' 
2177      paramd = indepvarname != "" 
2178      if not paramd and indepvararray is not None: 
2179          raise ValueError("Must supply independent variable name for "
 
2180                           "parameterized Pointset") 
2181      if paramd and indepvararray is None: 
2182          iv = [] 
2183      i = 0 
2184      labels = {} 
2185      for p in pointlist: 
2186          assert isinstance(p, Point), \
 
2187                 "pointlist argument must only contain Points" 
2188          if coordnames == []: 
2189              ptype = p.coordtype 
2190              pdim = p.dimension 
2191              coordnames = p.coordnames 
2192              xcoordnames = copy(coordnames) 
2193              if paramd and indepvararray is None: 
2194                  assert indepvarname in coordnames, \
 
2195                      "Independent variable name missing" 
2196                  del xcoordnames[xcoordnames.index(indepvarname)] 
2197              dv = {}.fromkeys(xcoordnames) 
2198              for c in xcoordnames: 
2199                  dv[c] = [] 
2200              if p.labels != {}: 
2201                  labels.update({0: p.labels}) 
2202                  i += 1 
2203          else: 
2204              # coerce ints to float types if mixed
 
2205              if compareNumTypes(ptype, int32): 
2206                  if compareNumTypes(p.coordtype, float64): 
2207                      ptype = float64 
2208                  elif compareNumTypes(p.coordtype, int32): 
2209                      pass 
2210                  else: 
2211                      raise TypeError("Type mismatch in points") 
2212              elif compareNumTypes(ptype, float64): 
2213                  if not compareNumTypes(p.coordtype, (float64, int32)): 
2214                      raise TypeError("Type mismatch in points") 
2215              else: 
2216                  raise TypeError("Type mismatch in points") 
2217              assert pdim == p.dimension, "Dimension mismatch in points" 
2218              if remain(coordnames,p.coordnames) != []: 
2219                  raise ValueError("Coordinate name mismatch in points") 
2220              if p.labels != {}: 
2221                  labels.update({i: p.labels}) 
2222                  i += 1 
2223          for c in xcoordnames: dv[c].append(p(c)) 
2224          if paramd and indepvararray is None: 
2225              iv.append(p(indepvarname)) 
2226      # submit data as array to maintain coordname ordering present in Points
 
2227      dim = len(xcoordnames) 
2228      ca = array([dv[c] for c in xcoordnames], ptype) 
2229      argDict = {'coordarray': ca,
 
2230                 'coordnames': xcoordnames,
 
2231                 'coordtype': ptype,
 
2232                 'labels': labels,
 
2233                 'norm': norm
 
2234                   } 
2235      if paramd: 
2236          if indepvararray is None: 
2237              indepvararray = array(iv, ptype) 
2238          argDict.update({'indepvarname': indepvarname,
 
2239                           'indepvararray': indepvararray,
 
2240                           'indepvartype': indepvartype}) 
2241      return Pointset(argDict)

2242  
 
2243  
 


2244 -def arrayToPointset(a, vnames=None, ia=None, iname=""):


2245      """Convert an array to a non-parameterized Pointset. The inclusion of an
 
2246      optional independent variable array creates a parameterized Pointset.
 
2247  
 
2248      Coordinate (and independent variable) names are optional: the defaults are
 
2249      the array indices (and 't' for the independent variable).
 
2250      """ 
2251      if rank(a) > 2: 
2252          raise ValueError("Cannot convert arrays of rank > 2") 
2253      if rank(a) == 0: 
2254          raise ValueError("Cannot convert arrays of rank 0") 
2255      if vnames is None: 
2256          vnames = [str(i) for i in range(shape(a)[0])] 
2257      else: 
2258          if len(vnames) != shape(a)[0]: 
2259              raise ValueError("Mismatch between number of coordinate names and"
 
2260                               " number of rows in array.\nCoordinates are "
 
2261                               "assumed to be the rows of the array") 
2262      if ia is None: 
2263          assert iname=="", ("Independent variable name must be none if no "
 
2264                             "independent variable array provided") 
2265          return Pointset({'coordarray': a,
 
2266                       'coordnames': vnames}) 
2267      else: 
2268          if iname == "": 
2269              iname = "t" 
2270          return Pointset({'coordarray': a,
 
2271                       'coordnames': vnames,
 
2272                       'indepvararray': ia,
 
2273                       'indepvarname': iname})

2274  
 


2275 -def exportPointset(thepointset, infodict, separator='   ',
 
2276                     precision=12, varvaldir='col',
 
2277                     ext='', append=False):


2278      """Export a pointset to a set of ASCII whitespace- (or
 
2279      user-defined character-) separated data files. Option to list each
 
2280      variable's data in rows ('across') or in columns ('down').
 
2281      Existing files of the same names will be overwritten, unless the
 
2282      'append' boolean option is set.
 
2283  
 
2284      NB. If the file extension argument 'ext' is present without a
 
2285      leading dot, one will be added.
 
2286  
 
2287      infodict should consist of: keys = filenames, values = tuples of
 
2288      pointset variable names to export.
 
2289      """ 
2290      assert varvaldir in ['col', 'row'], \
 
2291             "invalid variable value write direction" 
2292      # in order to avoid import cycles, cannot explicitly check that
 
2293      # thepointset is of type Pointset, because Points.py imports this file
 
2294      # (utils.py), so check an attribute instead.
 
2295      try: 
2296          thepointset.coordnames 
2297      except AttributeError: 
2298          raise TypeError("Must pass Pointset to this function: use "
 
2299                          "arrayToPointset first!") 
2300      infodict_usedkeys = [] 
2301      for key, info in infodict.iteritems(): 
2302          if isinstance(info, str): 
2303              infodict_usedkeys += [info] 
2304          elif info == []: 
2305              infodict[key] = copy.copy(thepointset.coordnames) 
2306              infodict_usedkeys.extend(thepointset.coordnames) 
2307          else: 
2308              infodict_usedkeys += list(info) 
2309      allnames = copy(thepointset.coordnames) 
2310      if thepointset._parameterized: 
2311          allnames.append(thepointset.indepvarname) 
2312      remlist = remain(infodict_usedkeys, allnames+range(len(allnames))) 
2313      if remlist != []: 
2314          print "Coords not found in pointset:", remlist 
2315          raise ValueError("invalid keys in infodict - some not present "
 
2316                           "in thepointset") 
2317      assert isinstance(ext, str), "'ext' extension argument must be a string" 
2318      if ext != '': 
2319          if ext[0] != '.': 
2320              ext = '.'+ext 
2321      if append: 
2322          assert varvaldir == 'col', ("append mode not supported for row"
 
2323                                       "format of data ordering") 
2324          modestr = 'a' 
2325      else: 
2326          modestr = 'w' 
2327      totlen = len(thepointset) 
2328      if totlen == 0: 
2329          raise ValueError("Pointset is empty") 
2330      for fname, tup in infodict.iteritems(): 
2331          try: 
2332              f = open(fname+ext, modestr) 
2333          except IOError: 
2334              print "There was a problem opening file "+fname+ext 
2335              raise 
2336          try: 
2337              if isinstance(tup, str): 
2338                  try: 
2339                      varray = thepointset[tup] 
2340                  except TypeError: 
2341                      raise ValueError("Invalid specification of coordinates") 
2342              elif isinstance(tup, int): 
2343                  try: 
2344                      varray = thepointset[:,tup].toarray() 
2345                  except TypeError: 
2346                      raise ValueError("Invalid specification of coordinates") 
2347              elif isinstance(tup, (list, tuple)): 
2348                  if alltrue([isinstance(ti,str) for ti in tup]): 
2349                      thetup=list(tup) 
2350                      if thepointset.indepvarname in tup: 
2351                          tix = thetup.index(thepointset.indepvarname) 
2352                          thetup.remove(thepointset.indepvarname) 
2353                      try: 
2354                          vlist = thepointset[thetup].toarray().tolist() 
2355                      except TypeError: 
2356                          raise ValueError("Invalid specification of coordinates") 
2357                      if len(thetup)==1: 
2358                          vlist = [vlist] 
2359                      if thepointset.indepvarname in tup: 
2360                          vlist.insert(tix, thepointset.indepvararray.tolist()) 
2361                      varray = array(vlist) 
2362                  elif alltrue([isinstance(ti,_int_types) for ti in tup]): 
2363                      try: 
2364                          varray = thepointset[:,tup].toarray() 
2365                      except TypeError: 
2366                          raise ValueError("Invalid specification of coordinates") 
2367                  else: 
2368                      raise ValueError("Invalid specification of coordinates") 
2369              else: 
2370                  f.close() 
2371                  raise TypeError("infodict values must be singletons or "
 
2372                                  "tuples/lists of strings or integers") 
2373          except IOError: 
2374              f.close() 
2375              print "Problem writing to file"+fname+ext 
2376              raise 
2377          except KeyError: 
2378              f.close() 
2379              raise KeyError("Keys in infodict not found in pointset") 
2380          if isinstance(precision, int): 
2381              assert precision > 0 
2382              ps = str(precision) 
2383          else: 
2384              raise TypeError("precision must be a positive integer") 
2385          if varvaldir == 'row': 
2386              savetxt(f, varray, '%.'+ps+'f', separator) 
2387          else: 
2388              savetxt(f, transpose(varray), '%.'+ps+'f', separator) 
2389          f.close()

2390  
 
2391  
 


2392 -def importPointset(xFileName, t=None, indices=None, sep=" ",
 
2393                     preamblelines=0):


2394      """Import ASCII format files containing data points.
 
2395      If the first row contains string names then the output
 
2396      will be a pointset, otherwise a numeric array.
 
2397  
 
2398      A dictionary is returned, with keys 'vararray' will point to the
 
2399      data. The 't' argument can specify one of several things:
 
2400  
 
2401      string: filename to read single-column of time values (same length as
 
2402              xFileName)
 
2403      sequence type: time values (same length as xFileName)
 
2404      integer: column in xFileName to treat as time data
 
2405  
 
2406      If used, this leads to and an additional key in the return
 
2407      dictionary where 't' points to the independent variable array.
 
2408  
 
2409      Specific columns can be selected for the variable data array by
 
2410      specifying a list of column indices in argument 'indices'.
 
2411  
 
2412      The separator used in the ASCII file can be specified by argument
 
2413      'sep' (defaults to single whitespace character).
 
2414  
 
2415      preamblelines (positive integer) specifies how many lines to skip before
 
2416      starting to read data (in case of preceding text) -- default 0.
 
2417      """ 
2418  
 
2419      if indices is None: 
2420          indices = [] 
2421      xFile = open(xFileName, 'r') 
2422      xFileStrList = xFile.readlines() 
2423      filelen = len(xFileStrList)-preamblelines 
2424      if filelen == 1 and '\r' in xFileStrList[0]: 
2425          # fix problem when no newlines picked up, only '\r'
 
2426          xFileStrList = xFileStrList[0].split('\r') 
2427          filelen = len(xFileStrList) 
2428      if filelen <= 1: 
2429          raise ValueError("Only 1 data point found in variables datafile") 
2430      x_dummy_all = xFileStrList[preamblelines].rstrip("\n") 
2431      x_dummy_vallist = filter(lambda s: s != '', x_dummy_all.split(sep)) 
2432      if t is None: 
2433          get_t = 0 
2434      elif isinstance(t, str): 
2435          tFileName = t 
2436          tFile = open(tFileName, 'r') 
2437          tFileStrList = tFile.readlines() 
2438          if len(tFileStrList)-preamblelines != filelen: 
2439              raise ValueError("Length of data and time files must be equal"
 
2440                             " -- are there any blank lines in the files?") 
2441          get_t = 1 
2442      elif isinstance(t, _seq_types): 
2443          if len(t) != filelen: 
2444              raise ValueError("Length of data file and t array must be "
 
2445                         "equal -- are there any blank lines in the files?") 
2446          tVals = t 
2447          get_t = 0 
2448      elif isinstance(t, _int_types): 
2449          # t represents column index to find time data in data file
 
2450          if t >= len(x_dummy_vallist) or t < 0: 
2451              raise ValueError("t index out of range") 
2452          get_t = 2 
2453      if indices == []: 
2454          if get_t == 2: 
2455              dim = len(x_dummy_vallist)-1 
2456              indices = remain(range(0,dim+1),[t]) 
2457          else: 
2458              dim = len(x_dummy_vallist) 
2459              indices = range(0,dim) 
2460      else: 
2461          dim = len(indices) 
2462          if get_t == 2: 
2463              if t in indices: 
2464                  raise ValueError("You specified column "+str(t)+" as time "
 
2465                      "data, but you have specified it as a data column in "
 
2466                      "indices argument") 
2467      # try to find variable names. if successful, start at row 1
 
2468      start = preamblelines 
2469      # replace unnecessary quote marks in strings
 
2470      test_line = [n.strip('"').strip("'") for n in \
 
2471              xFileStrList[preamblelines].lstrip(sep).lstrip(" ").rstrip("\n").rstrip("\r").split(sep)] 
2472      def is_float(vstr): 
2473          try: 
2474              val = float(vstr) 
2475          except ValueError: 
2476              return False 
2477          else: 
2478              return True

2479      if alltrue([not is_float(n) for n in test_line]): 
2480          # success
 
2481          start += 1 
2482          # replace any internal spaces with underscores, remove dots
 
2483          test_line = [n.replace(" ", "_").replace(".","") for n in test_line] 
2484          if get_t == 2: 
2485              t_name = test_line[t] 
2486              varnames = test_line[0:t]+test_line[t+1:] 
2487          else: 
2488              if get_t == 1: 
2489                  # try first line of t file
 
2490                  t_test = tFileStrList[0].lstrip(" ").rstrip("\n").rstrip("\r").replace(".","").replace(" ","_").strip('"').strip("'") 
2491                  if is_float(t_test): 
2492                      # already checked that file lengths were the same
 
2493                      raise ValueError("First line of t file shouldn't be a number") 
2494                  else: 
2495                      t_name = t_test 
2496              else: 
2497                  t_name = 't' 
2498              varnames = test_line 
2499      else: 
2500          t_name = 't' 
2501          varnames = None 
2502      tVals = zeros(filelen-start, float) 
2503      xVals = zeros([filelen-start, dim], float) 
2504      # read rest of file
 
2505      for i in xrange(filelen-start): 
2506          vLine = xFileStrList[i+start].rstrip("\n") 
2507          if vLine == '': 
2508              continue 
2509          vLineVals = filter(lambda s: s != '', vLine.split(sep)) 
2510          if get_t == 1: 
2511              # Additional left strip of space char in case sep is different
 
2512              tLine = tFileStrList[i+start].rstrip("\n").lstrip(sep).lstrip(" ") 
2513              if len(tLine.split(sep)) != 1: 
2514                  raise ValueError("Only one t value expected per line of"
 
2515                                     " datafile") 
2516              if tLine == '': 
2517                  continue 
2518              tVals[i] = float(tLine) 
2519          elif get_t == 2: 
2520              tVals[i] = float(vLineVals[t]) 
2521          try: 
2522              xLineVals = [vLineVals[ix] for ix in indices] 
2523          except IndexError: 
2524              print "Valid indices were: 0 -", len(vLineVals)-1 
2525              raise 
2526          if len(xLineVals) != dim: 
2527              raise ValueError("Exactly "+str(dim)+" values expected per "
 
2528                                 "line of datafile") 
2529          xVals[i] = array([float(xstr) for xstr in xLineVals], float) 
2530      xFile.close() 
2531      if get_t == 1: 
2532          tFile.close() 
2533      if get_t == 0: 
2534          if varnames is None: 
2535              return xVals 
2536          else: 
2537              # non-parameterized pointset
 
2538              return Pointset(dict(zip(varnames, xVals))) 
2539      else: 
2540          if varnames is None: 
2541              return {t_name: tVals, 'vararray': xVals.T} 
2542          else: 
2543              return Pointset(indepvardict={t_name: tVals},
 
2544                              coorddict=dict(zip(varnames,xVals.T))) 
2545  
 
2546  
 


2547 -def export_pointset_to_CSV(filename, pts):


2548      """Simple export that ignores all metadata in pts,
 
2549      including name, tags, norm, etc.
 
2550      Data is arranged by row only.
 
2551  
 
2552      Independent variable is first column, if it exists in pts.
 
2553      """ 
2554      import csv 
2555      outfile = open(filename, 'w') 
2556      writer = csv.writer(outfile) 
2557  
 
2558      # header row
 
2559      if pts._parameterized: 
2560          rows_header = [pts.indepvarname] + pts.coordnames 
2561      else: 
2562          rows_header = pts.coordnames 
2563      writer.writerow(rows_header) 
2564  
 
2565      # data rows
 
2566      if pts._parameterized: 
2567          for i in xrange(len(pts)): 
2568              writer.writerow([pts.indepvararray[i]] + list(pts.coordarray[:,i])) 
2569      else: 
2570          for i in xrange(len(pts)): 
2571              writer.writerow(pts.coordarray[:,i]) 
2572  
 
2573      outfile.close()

2574  
 
2575  
 


2576 -def mergePointsets(pts1, pts2):


2577      """Merges two pointsets into a new pointset, preserving (merging) any
 
2578      metadata in each.
 
2579  
 
2580      In particular, if each have different accuracy tolerances, the
 
2581      larger of the two will be used. If each have different 'checklevel' values,
 
2582      the larger of the two will be used. Point labels will be merged. Names
 
2583      will also be merged.
 
2584  
 
2585      If both are parameterized, their independent variable arrays
 
2586      must be identical. If only one is parameterized, the result will be
 
2587      too. The two pointsets must be identical in length.
 
2588  
 
2589      The norm associated with each pointset must be the same.
 
2590      """ 
2591      len1 = len(pts1) 
2592      len2 = len(pts2) 
2593      assert len1 == len2, "Pointsets must have equal length" 
2594      assert pts1._normord == pts2._normord, "Pointsets must use the same norm" 
2595      isparam1 = isparameterized(pts1) 
2596      isparam2 = isparameterized(pts2) 
2597      if isparam1 and isparam2: 
2598          assert pts1.indepvarname == pts2.indepvarname, \
 
2599                 "Parameterized pointsets must have identical independent variable names" 
2600          assert all(pts1.indepvararray == pts2.indepvararray), \
 
2601                 "Parameterized pointsets must have identical independent variable values" 
2602      common_coords = intersect(pts1.coordnames, pts2.coordnames) 
2603      for c in common_coords: 
2604          assert all(pts1[c] == pts2[c]), \
 
2605             "Pointsets must not share any coordinate names whose values are not identical" 
2606      args = {} 
2607      if isparam1 or isparam2: 
2608          if isparam1: 
2609              tvals = pts1.indepvararray 
2610              tname = pts1.indepvarname 
2611          else: 
2612              tvals = pts2.indepvararray 
2613              tname = pts2.indepvarname 
2614          args['indepvardict'] = {tname: tvals} 
2615      args['checklevel'] = max(pts1.checklevel, pts2.checklevel) 
2616      args['tolerance'] = max(pts1._abseps, pts2._abseps) 
2617      if name is None: 
2618          if pts1.name == "": 
2619              name1 = "<unnamed>" 
2620          else: 
2621              name1 = pts1.name 
2622          if pts2.name == "": 
2623              name2 = "<unnamed>" 
2624          else: 
2625              name2 = pts2.name 
2626          args['name'] = "Merged %s:%s" % (name1, name2) 
2627      coorddict = pts1.todict() 
2628      coorddict.update(pts2.todict()) 
2629      args['coorddict'] = coorddict 
2630      lab1 = deepcopy(pts1.labels) 
2631      lab2 = deepcopy(pts2.labels) 
2632      lab2.update(lab1) 
2633      args['labels'] = lab2 
2634      return Pointset(**args)

2635  
 
2636  
 


2637 -def padPointset(pts, pinterval, value_dict, eps=None):


2638      """Pad a pointset pts with values from value_dict over the interval given
 
2639      by pinterval (pair). For each side of the interval outside of the current independent
 
2640      variable domain of pts, two new points are added, one at the outer limit
 
2641      of the interval, and one a distance eps (default the abseps setting of pts)
 
2642      from the existing closest point in pts.
 
2643      """ 
2644      tlo, thi = pinterval 
2645      ts = pts.indepvararray 
2646      all_dict = value_dict.copy() 
2647      assert remain(value_dict.keys(), pts.coordnames) == [] 
2648      if eps is None: 
2649          eps = pts._abseps 
2650      if tlo < ts[0]: 
2651          all_dict['t'] = tlo 
2652          pts.insert(Point(coorddict=all_dict,
 
2653                           labels='pad')) 
2654          all_dict['t'] = ts[0]-eps 
2655          pts.insert(Point(coorddict=all_dict,
 
2656                           labels='pad')) 
2657      if thi > ts[-1]: 
2658          all_dict['t'] = ts[-1]+eps 
2659          pts.insert(Point(coorddict=all_dict,
 
2660                           labels='pad')) 
2661          all_dict['t'] = thi 
2662          pts.insert(Point(coorddict=all_dict,
 
2663                           labels='pad')) 
2664      return pts

2665
```

  


| Home | Trees | Indices | Help | PyDSTool |
| --- | --- | --- | --- | --- |

|  |  |
| --- | --- |
| Generated by Epydoc 3.0.1 on Fri May 4 15:24:17 2012 | http://epydoc.sourceforge.net |
